# Transmission patterns of COVID-19 in the mainland of China and the efficacy of different control strategies: a data- and model-driven study

**DOI:** 10.1186/s40249-020-00709-z

**Published:** 2020-07-06

**Authors:** Jian Zu, Miao-Lei Li, Zong-Fang Li, Ming-Wang Shen, Yan-Ni Xiao, Fan-Pu Ji

**Affiliations:** 1 School of Mathematics and Statistics, Xi’an Jiaotong University, Xi’an, 710049 China; 2 National & Local Joint Engineering Research Center of Biodiagnosis and Biotherapy, The Second Affiliated Hospital of Xi’an Jiaotong University, Xi’an, 710004 China; 3 Key Laboratory of Environment and Genes Related to Diseases, Xi’an Jiaotong University, Ministry of Education of China, Xi’an, 710061 China; 4 School of Public Health, Health Science Center, Xi’an Jiaotong University, Xi’an, 710061 China; 5 Department of Infectious Diseases, The Second Affiliated Hospital of Xi’an Jiaotong University, 157 Xi Wu Road, Xi’an, 710004 Shaanxi Province PR China

**Keywords:** SARS-CoV-2, COVID-19, Transmission dynamics, Prevalence, Effective reproduction number, Intervention strategy

## Abstract

**Background:**

The coronavirus disease 2019 (COVID-19) outbreak has seriously endangered the health and lives of Chinese people. In this study, we predicted the COVID-19 epidemic trend and estimated the efficacy of several intervention strategies in the mainland of China.

**Methods:**

According to the COVID-19 epidemic status, we constructed a compartmental model. Based on reported data from the National Health Commission of People’s Republic of China during January 10–February 17, 2020, we estimated the model parameters. We then predicted the epidemic trend and transmission risk of COVID-19. Using a sensitivity analysis method, we estimated the efficacy of several intervention strategies.

**Results:**

The cumulative number of confirmed cases in the mainland of China will be 86 763 (95% *CI:* 86 067*–*87 460) on May 2, 2020. Up until March 15, 2020, the case fatality rate increased to 6.42% (95% *CI:* 6.16*–*6.68%). On February 23, 2020, the existing confirmed cases reached its peak, with 60 890 cases (95% *CI:* 60 350*–*61 431). On January 23, 2020, the effective reproduction number was 2.620 (95% *CI:* 2.567*–*2.676) and had dropped below 1.0 since February 5, 2020. Due to governmental intervention, the total number of confirmed cases was reduced by 99.85% on May 2, 2020. Had the isolation been relaxed from February 24, 2020, there might have been a second peak of infection. However, relaxing the isolation after March 16, 2020 greatly reduced the number of existing confirmed cases and deaths. The total number of confirmed cases and deaths would increase by 8.72 and 9.44%, respectively, due to a 1-day delayed diagnosis in non-isolated infected patients. Moreover, if the coverage of close contact tracing was increased to 100%, the cumulative number of confirmed cases would be decreased by 88.26% on May 2, 2020.

**Conclusions:**

The quarantine measures adopted by the Chinese government since January 23, 2020 were necessary and effective. Postponing the relaxation of isolation, early diagnosis, patient isolation, broad close-contact tracing, and strict monitoring of infected persons could effectively control the COVID-19 epidemic. April 1, 2020 would be a reasonable date to lift quarantine in Hubei and Wuhan.

## Background

Emerging infectious diseases are a major challenge for global public health. In December 2019, a cluster of cases of pneumonia caused by 2019-nCoV (later named as severe acute respiratory syndrome-coronavirus-2, SARS-CoV-2) was reported in Wuhan, China [1]. More than 210 countries/territories reported coronavirus disease 2019 (COVID-19) in early May. On January 23, 2020 Wuhan went into lockdown to limit population movement and reduce human-to-human transmission. The pneumonia outbreak caused by SARS-CoV-2 was impacting people throughout the country. On January 30, 2020 the World Health Organization (WHO) declared that the pneumonia outbreak associated with SARS-CoV-2 was a public health emergency of international concern. Accordingly, the outbreak of COVID-19 had seriously endangered the lives and health of the Chinese people and brought heavy economic burden to the country. The accurate prediction of the COVID-19 epidemic trend, as well as an accurate estimation of the efficacy of prevention and control strategies, are major health challenges that need to be addressed immediately.

COVID-19 can be transmitted through body fluid droplets, contact, and respiratory aerosols of different sizes spread at close range [1, 2]. The incubation period of SARS-CoV-2 is 3–7 days, with an average of 5.2 days [1, 2]. Since SARS-CoV-2 is a single-stranded RNA virus that is susceptible to mutation, the general population is susceptible to this new coronavirus [3–5]. There is currently no SARS-CoV-2 vaccine available, and the development of a new vaccine could take several years [6]. As a result, COVID-19 has spread rapidly throughout the mainland of China. According to the report of the National Health Commission of China, as of February 18, 2020 there have been 74 185 confirmed cases (including 11 977 severe cases), 14 376 cured cases, and 2004 deaths, posing a serious threat to public health and economic development [7, 8].

In order to prevent and control COVID-19, the Chinese government has implemented a series of strict prevention and control measures, including strict close-contact tracing, quarantining of individuals with suspected infection, strict immigrant monitoring, etc. Based on reported data before January 23, 2020, earlier studies predicted COVID-19 prevalence and estimated the efficacy of interventional strategies [9–12]. However, few studies accurately predicted the peak time and ultimate extent of infection, and many studies had not used the reported data in a comprehensive manner. There was also no systematic evaluation of the efficacy of different prevention and control strategies, especially with respect to when relaxed isolation should be implemented.

This study aims to accurately predict COVID-19 prevalence in the mainland of China and to evaluate the impact of isolation intensity, delayed diagnosis, the external input of free infected persons, and the increased coverage of close-contact tracing on the SARS-CoV-2 transmission trend and case fatality rate.

## Methods

### Reported data from the National Health Commission of China

The reported COVID-19 data used in this study were collected primarily from the National Health Commission of China [7], including the cumulative numbers of confirmed, suspected cases and deaths, and the numbers of existing suspected cases, recovered cases, close-contacts under medical observation, and existing confirmed cases from January 10, 2020 to Febuary 17, 2020 (Supplementary p. 1–4). All of these data were used to estimate the initial values and parameters of the following model.

### Model description

According to the SARS-CoV-2 transmission mechanism, the actual process of clinical diagnosis, and the prevention and control measures, such as quarantine, isolation and treatment, we developed a continuous susceptible-exposed-infectious-suspected-confirmed-recovered (SEIPQR) compartmental model of COVID-19 transmission at the population level [1, 9, 10, 13, 14]. Specifically, we took into account two non-pharmaceutical interventions—quarantine and close-contact tracing. We divided the total population into the following eight compartments: susceptible individuals in the free environment (*S*); quarantined susceptible individuals who had close-contact with confirmed or suspected individuals (*S*_*q*_); free exposed (latent) individuals (*E*); isolated exposed individuals (*E*_*q*_); isolated suspected individuals (*P*), who were either exposed individuals or those with similar symptoms to COVID-19 cases; undiagnosed and non-isolated infectious individuals (*I*); confirmed and isolated infectious individuals (*Q*); and recovered individuals (*R*). For simplicity, we made the following assumptions:

(i) The total population was constant (1.4 billion) and was homogeneously distributed;
(ii) The susceptibility to infection for susceptible individuals in the free environment was the same.

**Fig. 1:**
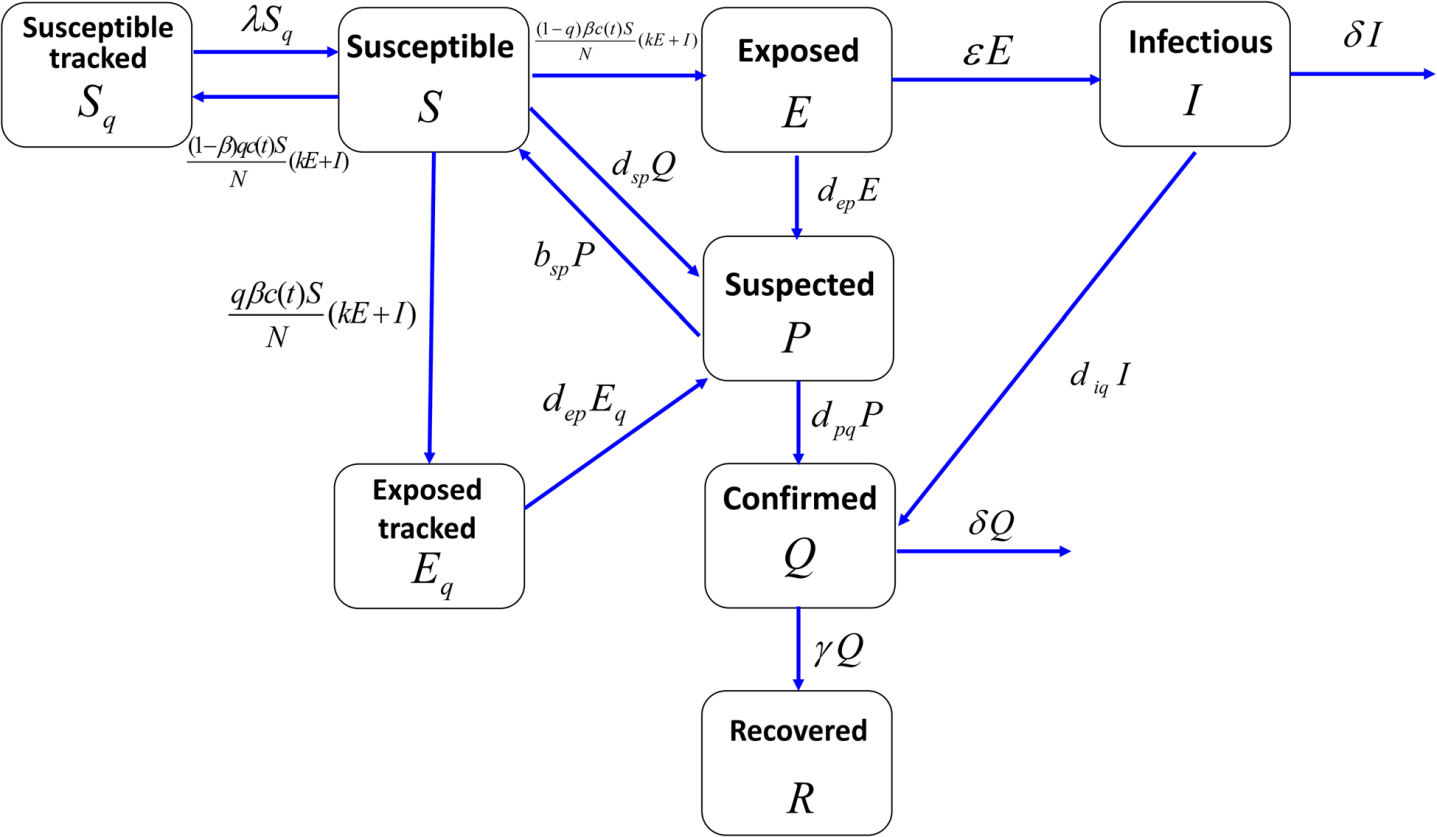
Flow chart of COVID-19 transmission model

Using the above assumptions, the transfer relationships between the eight compartments was shown in Fig. 1. Due to close-contact tracing, we assumed that a proportion (*q*) of individuals was quarantined, depending on whether or not they were exposed to SARS-CoV-2, and that quarantined individuals either moved to compartment *E*_*q*_ or *S*_*q*_. The other proportion (1 − *q*) of exposed individuals who were missed by contact-tracing were moved to non-isolated compartment *E*. We assumed that both non-isolated exposed individuals and infectious individuals were infectious. The relative transmission strength of exposed individuals to infectious individuals was *k*, the transmission probability was *β*, and the contact rate was *c*(*t*). Quarantined susceptible individuals returned to the free susceptible class at a rate of *λ*. Latent individuals progressed to the undiagnosed infectious compartment at a rate of *ε*. Non-isolated infectious individuals were diagnosed and confirmed at a rate of *d*_*iq*_ or died due to the disease at a rate of *δ*. We assumed that diagnosed and confirmed individuals were strictly isolated and could not further infect others. Traced and free exposed individuals were diagnosed as suspected cases at a rate of *d*_*ep*_. Suspected cases might be misdiagnosed, and the number of misdiagnosed individuals entering the *P* class was *d*_*sp*_*Q*. Misdiagnosed suspected cases returned to the susceptible class at a rate of *b*_*sp*_. Suspected individuals were further diagnosed and confirmed at a rate of *d*_*pq*_. Confirmed cases recovered at a rate of *γ*, or died from the disease at a rate of *δ*.

The introduction of compartment *P* was necessary and more in line with the clinical diagnostic standard. This is because there is a substantial number of individuals in compartment *P* without SARS-CoV-2 virus that were misdiagnosed as possible COVID-19 patients. Based on the above analysis and Fig. 1, the continuous SEIPQR model of COVID-19 transmission at the population level was given by the following equations:

1 $$ {\displaystyle \begin{array}{l} dS/ dt=-\frac{\left(1-q\right)\beta c(t)S}{N}\left( kE+I\right)-\frac{\left(1-\beta \right) qc(t)S}{N}\left( kE+I\right)-\frac{q\beta c(t)S}{N}\left( kE+I\right)-{d}_{sp}Q+{b}_{sp}P+\lambda {S}_q,\\ {} dS{}_q/ dt=\frac{\left(1-\beta \right) qc(t)S}{N}\left( kE+I\right)-\lambda S{}_q,\\ {} dE/ dt=\frac{\left(1-q\right)\beta c(t)S}{N}\left( kE+I\right)-\varepsilon E-{d}_{ep}E,\\ {}{dE}_q/ dt=\frac{q\beta c(t)S}{N}\left( kE+I\right)-{d}_{ep}{E}_q,\\ {} dI/ dt=\varepsilon E-{d}_{iq}I-\delta I,\\ {} dP/ dt={d}_{sp}Q+{d}_{ep}{E}_q+{d}_{ep}E-{b}_{sp}P-{d}_{pq}P,\\ {} dQ/ dt={d}_{pq}P-\gamma Q-\delta Q+{d}_{iq}I,\\ {} dR/ dt=\gamma Q.\end{array}} $$

Here, *N* represents the total population size, which we assumed to be 1.4 billion, for simplicity.

In order to more comprehensively use the reported data to estimate unknown parameters, we further derived the equations of the cumulative number of confirmed cases (*H*), cumulative number of suspected cases (*Y*), and cumulative number of deaths (*Z*), which were described by the following eq. (2):

2 $$ {\displaystyle \begin{array}{l} dH/ dt={d}_{iq}I+{d}_{pq}P,\\ {} dY/ dt={d}_{ep}E+{d}_{ep}{E}_q+{d}_{sp}Q,\\ {} dZ/ dt=\delta I+\delta Q.\end{array}} $$

The major difference with published studies was that models (1) and (2) comprehensively considered the role of various preventative and control measures, and distinguished the suspected cases compartment, which was more in line with the actual clinical diagnostic process. Based on this model, we can make full use of all the information from reported data (seven types of data) to estimate unknown parameters and discuss the comprehensive effectiveness of different control measures.

By using the next-generation matrix method [10, 15], we obtained the effective reproduction number of model (1), which was given by eq. (3).

3 $$ {R}_e(t)=\left[\frac{\left(1-q\right)\beta c(t) kS(0)}{2N\left(\varepsilon +{d}_{ep}\right)}+\frac{1}{2}\sqrt{{\left(\frac{\left(1-q\right)\beta c(t) kS(0)}{N\left(\varepsilon +{d}_{ep}\right)}\right)}^2+4\frac{\left(1-q\right)\beta c(t)S(0)}{N\left({d}_{iq}+\delta \right)}\frac{\varepsilon }{\left(\varepsilon +{d}_{ep}\right)}}\right]\frac{S(t)}{N} $$

### Initial conditions and input parameters

We chose January 23, 2020 as the initial time for models (1) and (2) because Wuhan was closed to outside entry on this date, which is also when the Chinese government began to implement strict isolation and control measures. The initial values of diagnosed individuals (*Q*(0)), recovered individuals (*R*(0)), cumulative confirmed cases (*H*(0)), cumulative suspected cases (*Y*(0))*,* and cumulative deaths (*Z*(0)) were given according to the reported data. Since the average SARS-CoV-2 incubation period was 5.2 days, the transfer rate of *ε* from compartment *E* to compartment *I* was set to 1/5.2 [2]. Since traced susceptible individuals had to be quarantined for 14 days, the transfer rate from compartment *S*_*q*_ to compartment *S* was 1/14 [16]. In addition, with the implementation of various preventative and control measures, and in consideration of public safety, people would consciously restrict their behavior and pay more attention to daily health and prevention, which might reduce the contact rate with infected or exposed individuals. Therefore, after January 23, 2020, due to the strict control measures implemented by the Chinese government, we assumed that the contact rate decreased exponentially [16, 17]:

4 $$ c(t)={c}_1+{c}_2\exp \left(-{c}_3t\right) $$

Other parameters and initial values in models (1) and (2) were required for estimations using the least square method and Markov Chain Monte Carlo (MCMC) approach [18, 19]. The initial values and specific estimated parameters were listed in Table 1. Specifically, in order to overcome the problem of model overfitting, we collected seven different data to fit the models (1) and (2), and estimated the unknown parameters, including the cumulative numbers of confirmed, suspected cases and deaths, and the numbers of existing suspected cases, recovered cases, close-contacts under medical observation, and existing confirmed cases from January 23, 2020 to February 17, 2020 (Table S1, Supplementary p. 2). First, we estimated a set of results using the least square method by setting the iteration number to 100 000. We then used these results as initial values when applying the MCMC method by setting the iteration number to 8000 and the first 6000 iterations as burn-in periods. Finally, we obtained the initial values and estimated parameters in model (1). We listed the description of parameters, variables, and their values, as well as the corresponding 95% confidence intervals (*CI*s) in Table 1. In order to verify the validation of the model and estimated parameters, we further compared the model estimated values with the seven different reported data sets in Table S1, where the root mean square error was 2096. This comparison suggested that the estimated values by model were in very good agreement with real reported data, models (1) and (2), and that the estimated parameter values can be used to predict the future development trend of COVID-19 in the mainland of China (Supplementary p. 5–7). MATLAB codes (MATLAB. (2010). *version 7.10.0 (R2010a)*. Natick, Massachusetts: The MathWorks Inc.) of the least square and MCMC approaches were included in the supplementary material (Supplementary p. 23–34).

**Table 1:** Parameters and initial conditions description for models (1) and (2)

Parameter	Meaning	Value	95% *CI*	Reference
*q*	Quarantine rate of close contacts	0.2653	(0.2651, 0.2655)	Estimated
*β*	Transmission rate	0.0792	(0.0788, 0.0796)	Estimated
*k*	Relative transmission strength of exposed individuals to the undiagnosed infectious individuals	0.5825	(0.5823, 0.5827)	Estimated
*b*_*sp*_	Transfer rate of suspected individuals to the unquarantined susceptible class	0.0867	(0.0866, 0.0867)	Estimated
*d*_*iq*_	Transfer rate of undiagnosed infectious individuals to the confirmed class	0.1333	(0.1277, 0.1390)	Estimated
*d*_*sp*_	Transfer rate of unquarantined susceptible individuals to the suspected class	3.8124e-05	(0.3573e-04, 0.4051e-04)	Estimated
*d*_*ep*_	Transfer rate of traced and free latent individuals to the suspected class	0.3106	(0.3105, 0.3106)	Estimated
*d*_*pq*_	Transfer rate of suspected individuals to the confirmed class	0.1130	(0.1129, 0.1130)	Estimated
***c***(*t*)	Contact rate	*c*(*t*) = *c*_1_ + *c*_2_ exp(−*c*_3_*t*)		Estimated
	*c*_1_Minimum contact rate	0.2362	(0.2225, 0.2499)	
	*c*_2_Adjustment coefficient	20.0877	(20.0092, 20.1662)	
	*c*_3_Exponential decline rate	0.1249	(0.1238, 0.1259)	
*δ*	Death rate due to infection	0.0022	(0.0021, 0.0022)	Estimated
*γ*	Recovery rate	0.0154	(0.0149, 0.0159)	Estimated
*λ*	Release rate of traced susceptible individuals to the unquarantined susceptible class	1/14	–	18
*ε*	Transfer rate of non-isolated latent individuals to the undiagnosed infectious class	1/5.2	–	4
*S*(0)	Initial value of susceptible individuals in the free environment	1.3875e+09	(1.3873e+09, 1.3877e+09)	Estimated
*S*_*q*_(0)	Initial value of quarantined susceptible individuals	781	(777, 784)	Estimated
*E*(0)	Initial value of non-isolated latent individuals	1.170e+03	(1.168e+03, 1.172e+03)	Estimated
*E*_*q*_(0)	Initial value of isolated latent individuals	2.653e+03	(2.653e+03, 2.654e+03)	Estimated
*I*(0)	Initial value of undiagnosed infectious individuals	794	(792, 796)	Estimated
*P*(0)	Initial value of suspected individuals	1.888e+03	(1.886e+03, 1.890e+03)	Estimated
***Q***(*0*)	Initial value of confirmed and isolated individuals	771	–	Reported data
***R***(*0*)	Initial value of recovered individuals	34	–	Reported data
***H***(*0*)	Initial value of cumulative confirmed cases	830	–	Reported data
***Y***(*0*)	Initial value of cumulative suspected cases	1072	–	Reported data
***Z***(*0*)	Initial value of cumulative deaths	25	–	Reported data
*N*	Total population in the mainland of China	1 400 000 000	–	Reported data

In order to discuss the impact of China’s strict prevention and control measures, we compared and analysed two scenarios. In one, no prevention and control measures had been taken since January 10, 2020; in the other, strict prevention and control measures had been taken since January 23, 2020. Without any control measures, we reduced three compartments in model (1) and obtained a new susceptible-exposed-infectious-confirmed-recovered (SEIQR) model (S1) (Supplementary p. 12). If there were no prevention and control measures since January 10, 2020, the population migration would gradually increase during the Spring Festival in China, so the contact rate would peak during the Chinese Spring Festival. However, with the gradual outbreak of the epidemic, people would be expected to respond to the disease, possibly consciously reducing their contact behaviors; as such, after the Spring Festival, the contact rate would gradually decrease. Therefore, we assumed that the exposure rate of *c*(*t*) would obey a normal distribution, which would have reached its maximum during the Spring Festival (see Formula (S3), Supplementary p. 12). Based on the cumulative confirmed cases, cumulative deaths and cured cases reported by the National Health Commission of China from January 10–22, 2020, we re-estimated the initial values and parameters of models (S1) and (S2) (Supplementary p. 12–14), which were shown in Table S4 (Supplementary p. 13). We also compared the estimated values by models (S1) and (S2) with the three different reported data in Figure S4 (Supplementary p. 14), which served as a validation of the model.

## Results

### COVID-19 epidemic trend in the mainland of China

**Fig. 2:**
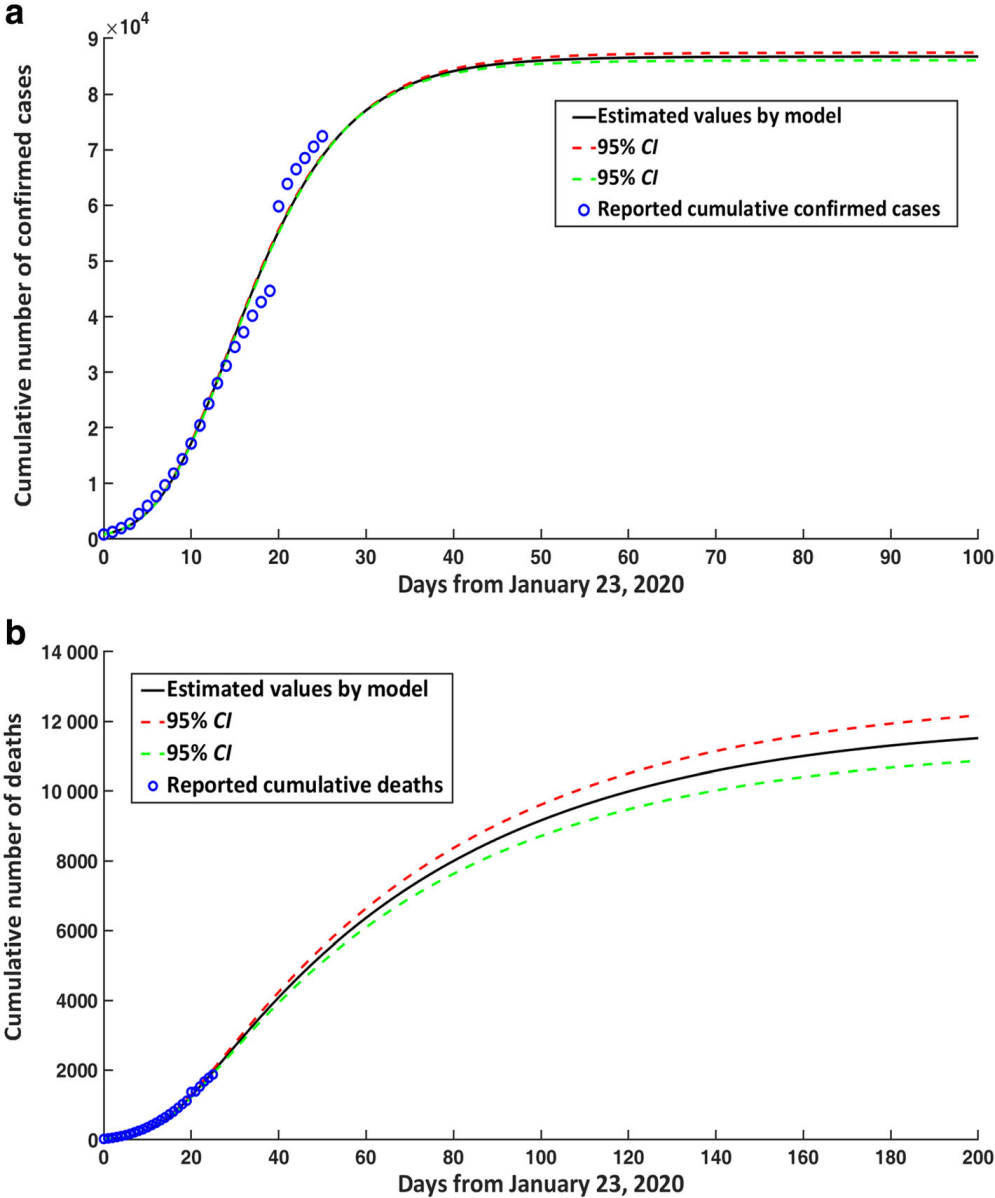
COVID-19 epidemic trends in the mainland of China for cumulative cases over time. **a** Confirmed cases. **b** Deaths. *CI*: confidence interval; COVID-19: Corona virus disease 2019

The final cumulative number of confirmed cases in the mainland of China would reach 86 763 (95% *CI:* 86 067–87 460) on May 2, 2020 (Fig. 2a). The cumulative number of confirmed cases would stabilize from April 1, 2020, which indicated that the number of new confirmed cases every day was minimal. Since March 13, 2020, the number of new confirmed cases would be less than 100 every day, and from April 1, 2020, the number of new confirmed cases would be less than 10 every day. The number of new daily confirmed cases began to decline since February 8, 2020. Up until March 15th, the cumulative number of deaths in the mainland of China would reach 5535 (95% *CI:* 5308–5763). Overall mortality rate due to disease was approximately 6.42% (95% *CI:* 6.16–6.68%) (Fig. 2b).

**Fig. 3:**
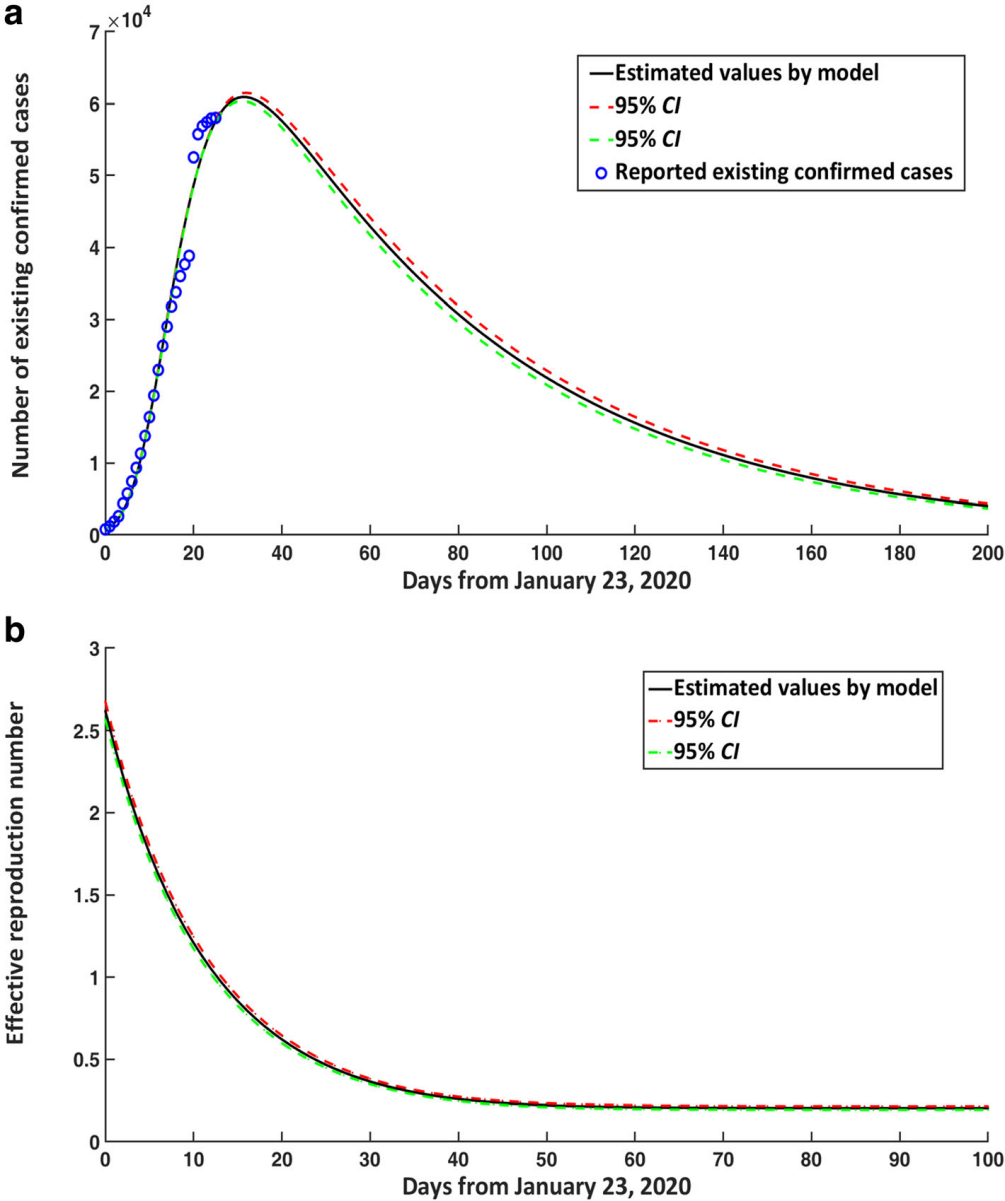
COVID-19 epidemic trends in the mainland of China for existing confirmed cases and effective reproduction number over time. **a** Number of existing confirmed cases in the mainland of China. **b** Estimated effective reproduction number. *CI*: confidence interval; COVID-19: Corona virus disease 2019

The number of existing confirmed cases in the mainland of China would reach its peak around February 23, 2020, and the number would reach 60 890 cases (95% *CI:* 60 350–61 431) (Fig. 3a). After the peak, the number of existing confirmed cases would decline slowly, following a chi-square distribution approximately.

Substituting the initial and parameter values in Table 1 into eq. (3), we can see that the effective reproduction number of COVID-19 on January 23, 2020 was about 2.620 (95% *CI:* 2.567–2.676). On February 5, 2020, the effective reproduction number had dropped below 1.0, which suggested that the number of new infections would gradually decline from February 5, 2020 (Fig. 3b).

### Impact of different prevention and control strategies on the COVID-19 epidemic trend

**Fig. 4:**
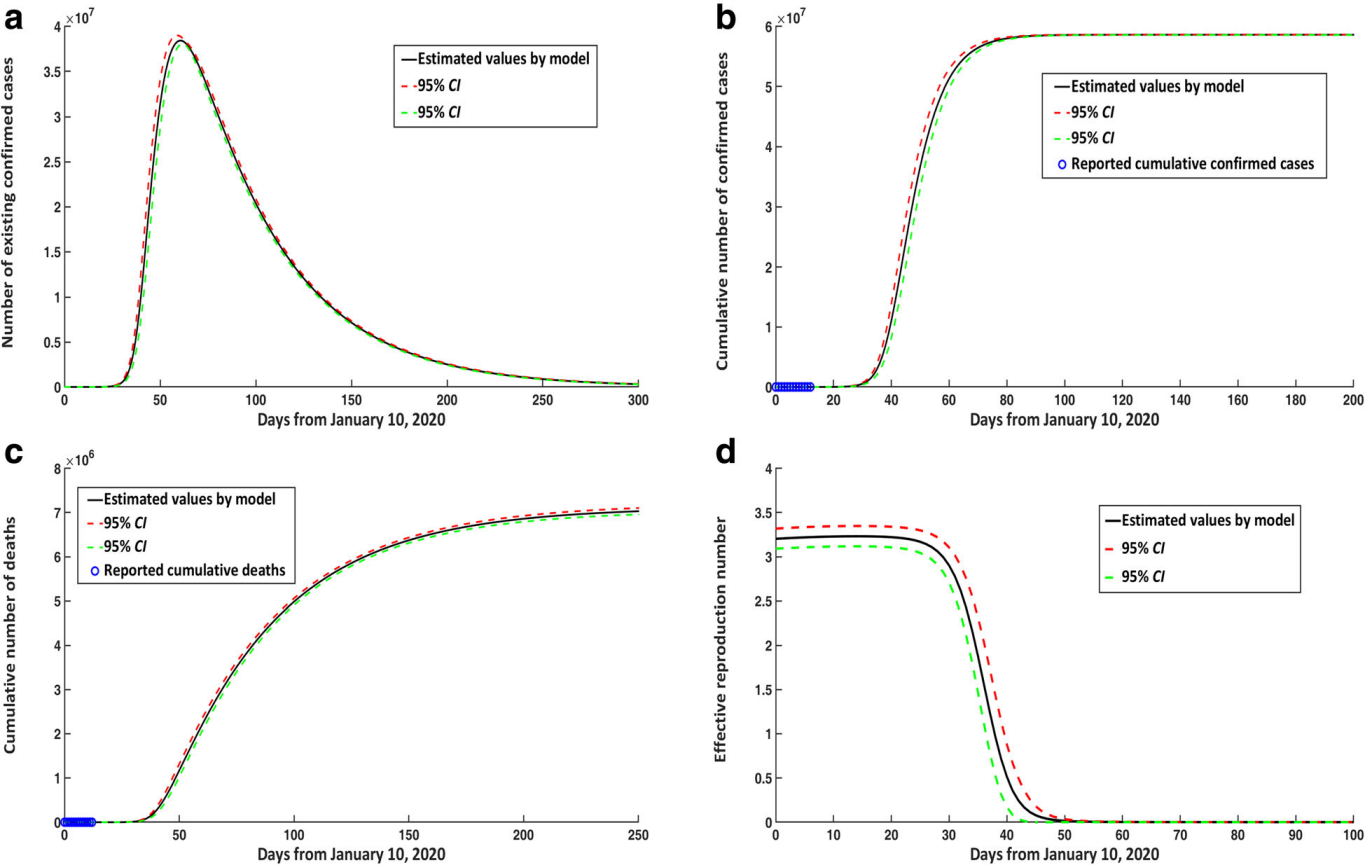
COVID-19 epidemic trends in the mainland of China over time without any control measures from January 10, 2020. **a** Number of existing confirmed cases. **b** Cumulative number of confirmed cases. **c** Cumulative number of deaths. **d** Estimated effective reproduction number without any control measures from January 10, 2020. *CI*: confidence interval; COVID-19: Corona virus disease 2019

Before January 23, 2020, because there were no quarantine and close-contact tracing, so we reduced three compartments in model (1) and obtained a new SEIQR model (S1) (Supplementary p. 12). Based on the new model (S1), we found that there were no interventional measures that began on January 10, 2020, then the peak time of existing confirmed cases would have appeared around March 11, 2020, and the number at peak time would have reached 38 425 800, representing an increase of 38 364 910 cases compared with the current situation (Fig. 4a). Similarly, the cumulative number of confirmed cases and deaths in the mainland of China would have reached 58 613 197 and 7 117 271, respectively (Fig. 4b, c). In addition, the effective reproduction number would have reached 3.231 (95% *CI:* 3.117–3.348) on January 23, 2020 (Fig. 4d). The cumulative number of confirmed cases, existing confirmed cases at the peak time, and deaths would be reduced by 99.85, 99.84, and 99.84%, respectively, compared with the quarantine and prevention measures implemented by the Chinese government since January 23, 2020.

#### Impact of relaxing isolation

**Fig. 5:**
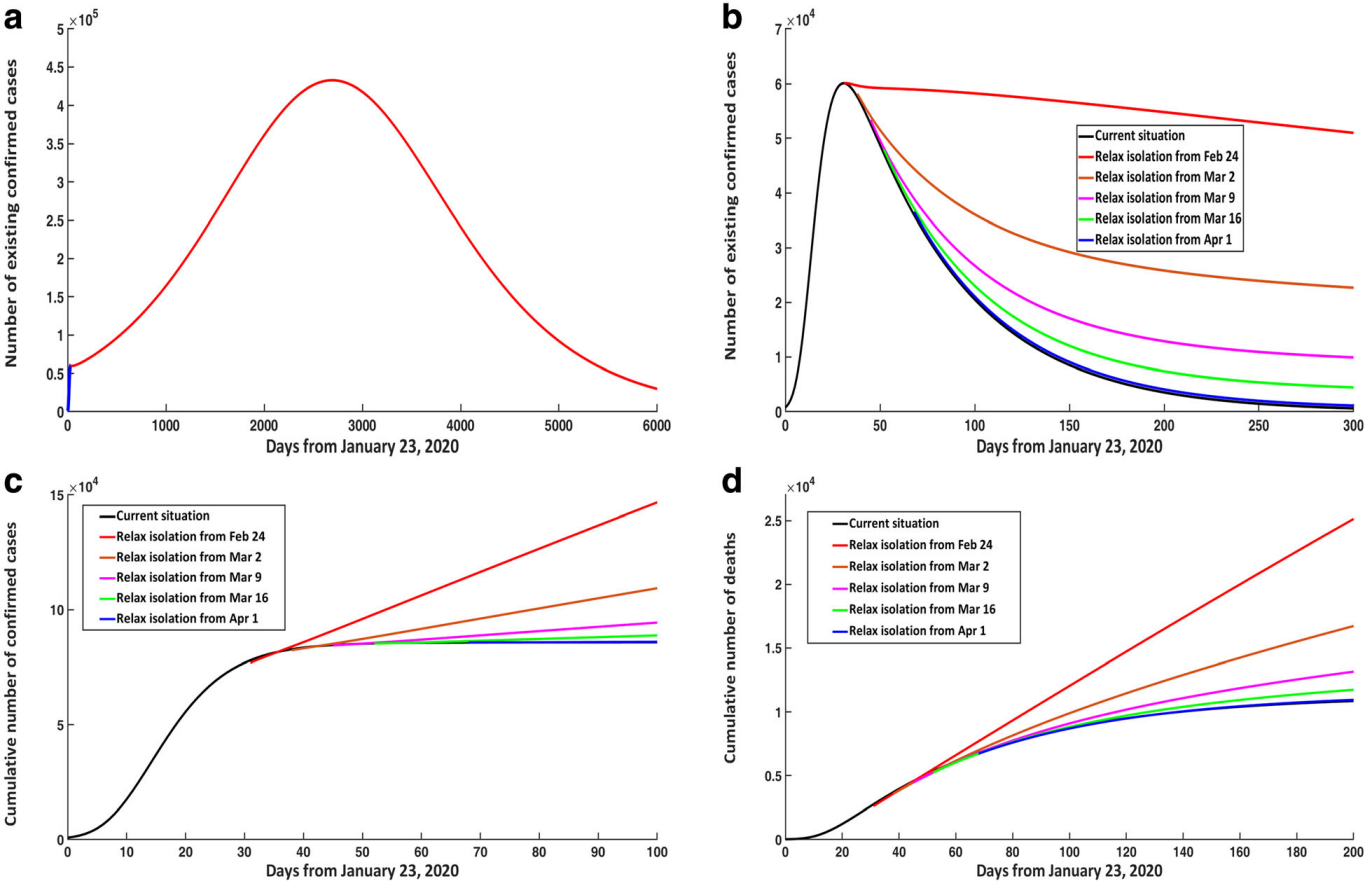
Impact of relaxing isolation at different times on COVID-19 epidemic trends in the mainland of China. **a** Number of existing confirmed cases when *c* = 4.394. **b** Number of existing confirmed cases when *c* = 4.334. **c** Cumulative number of confirmed cases when *c* = 4.334. **d** Cumulative number of deaths when *c* = 4.334. COVID-19: Corona virus disease 2019

When discussing the impact of relaxing isolation, we assumed that the contact rate would no longer exponentially decline as in eq. (4) but would instead become a constant *c*. If the quarantine was relaxed from February 24, 2020 and the contact rate satisfied *c* > 4.356, then there would be a second peak of infection. Particularly, when *c* = 4.394, the number of existing confirmed cases would reach 433 100 at the time of the second peak on June 11, 2020 (Fig. 5a).

However, if the quarantine was relaxed from February 24, 2020 and the contact rate satisfied *c* ≤ 4.356, then the number of existing confirmed cases would continue to decline but the downward trend would be slower (when *c* = 4.334; see Fig. 5b). In Fig. 5, panels B and C, compared with the current situation, it was evident that when *c* = 4.334, if isolation was relaxed on February 24, March 2, March 9, March 16, and April 1, 2020, respectively, then on April 30, 2020, the number of existing confirmed cases would increase by 174.56%, 71.79%, 28.54%, 11.59%, and 2.59%, respectively; the cumulative number of confirmed cases would increase by 68.80%, 26.49%, 9.63%, 3.36%, and 0.26%, respectively.

Similarly, if the quarantine was relaxed on February 24, 2020, and the contact rate satisfied *c* = 4.394, then the cumulative number of deaths would reach 12 100 on April 30, 2020, representing an increase of 39.95% compared with the current situation. When *c* = 4.334, compared with the current situation, if the quarantine was relaxed from February 24, March 2, March 9, March 16 and April 1, 2020, respectively, then on April 30, 2020, the cumulative number of deaths would increase by 38.33%, 12.56%, 3.79%, 1.29%, and 0.48%, respectively (Fig. 5d).

#### Impact of delayed diagnosis

**Fig. 6:**
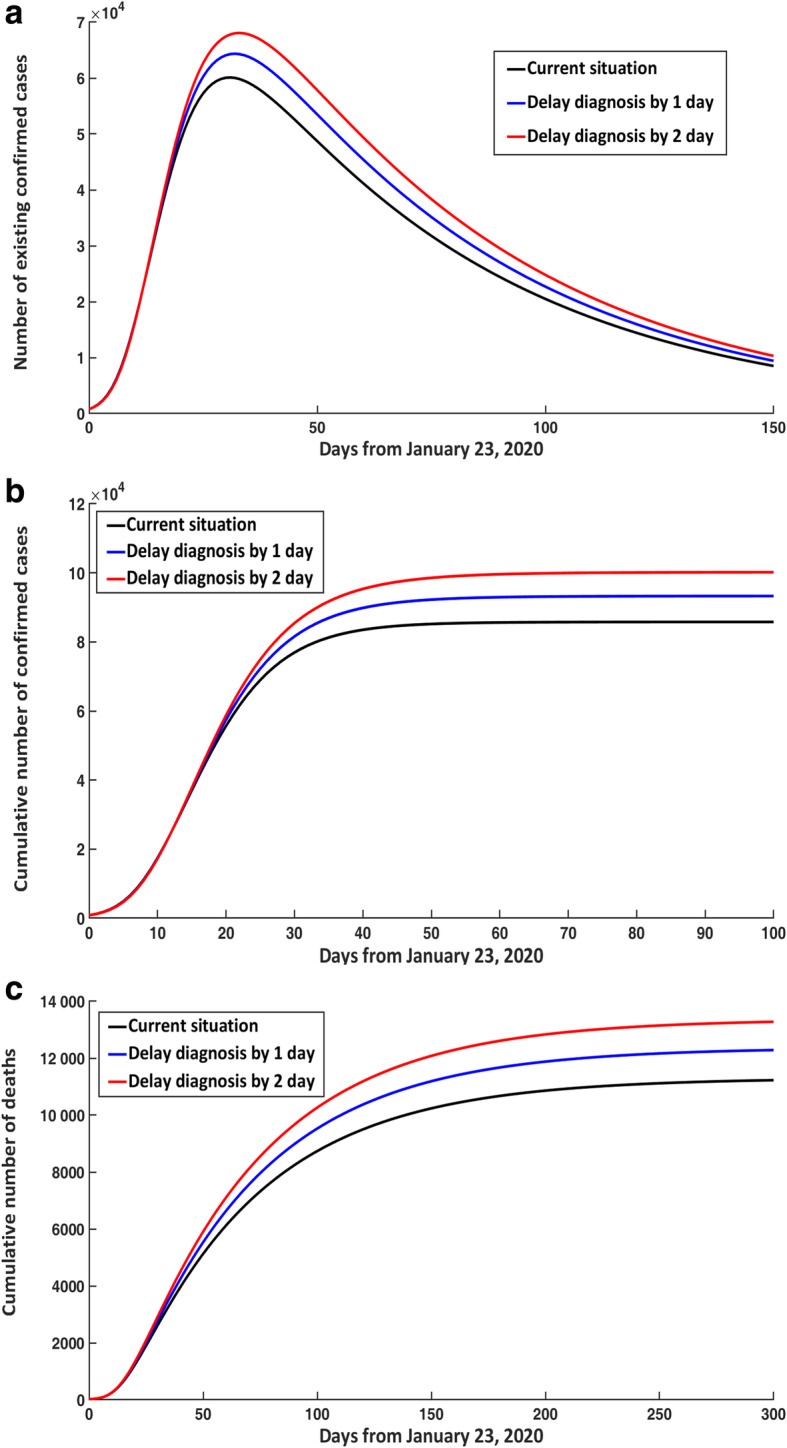
Effect of delayed diagnosis in non-isolated infectious individuals on COVID-19 epidemic trends. **a** Number of existing confirmed cases. **b** Cumulative number of confirmed cases. **c** Cumulative number of deaths. COVID-19: Corona virus disease 2019

If infectious individuals in the free environment had a delayed diagnosis by 1 day after January 23, 2020, the transfer rate of undiagnosed infectious individuals to the confirmed class would become *d*_*iq*_ = 0.1176. Therefore, the number of existing confirmed cases at peak time (February 24, 2020) and the total number of confirmed cases would increase by 4250 and 7480, respectively, which would correspond to an increase of 7.07% and 8.72% (Fig. 6a, b). If the diagnosis was delayed by 2 days, with a *d*_*iq*_ = 0.1052, the number of existing confirmed cases at peak time (February 25, 2020) and the total number of confirmed cases would increase by 7970 and 14 410, respectively, corresponding to an increase of 13.26% and 16.80%. The peak time would then be slightly delayed due to delayed diagnosis.

Similarly, if infectious individuals in the free environment had a delayed diagnosis for 1 or 2 days, the cumulative number of deaths would increase by 1060 and 2050, corresponding to an increase of 9.44% and 18.26%, respectively (Fig. 6c).

#### Impact of external input of infected persons and quarantined rate (*q*)

**Fig. 7:**
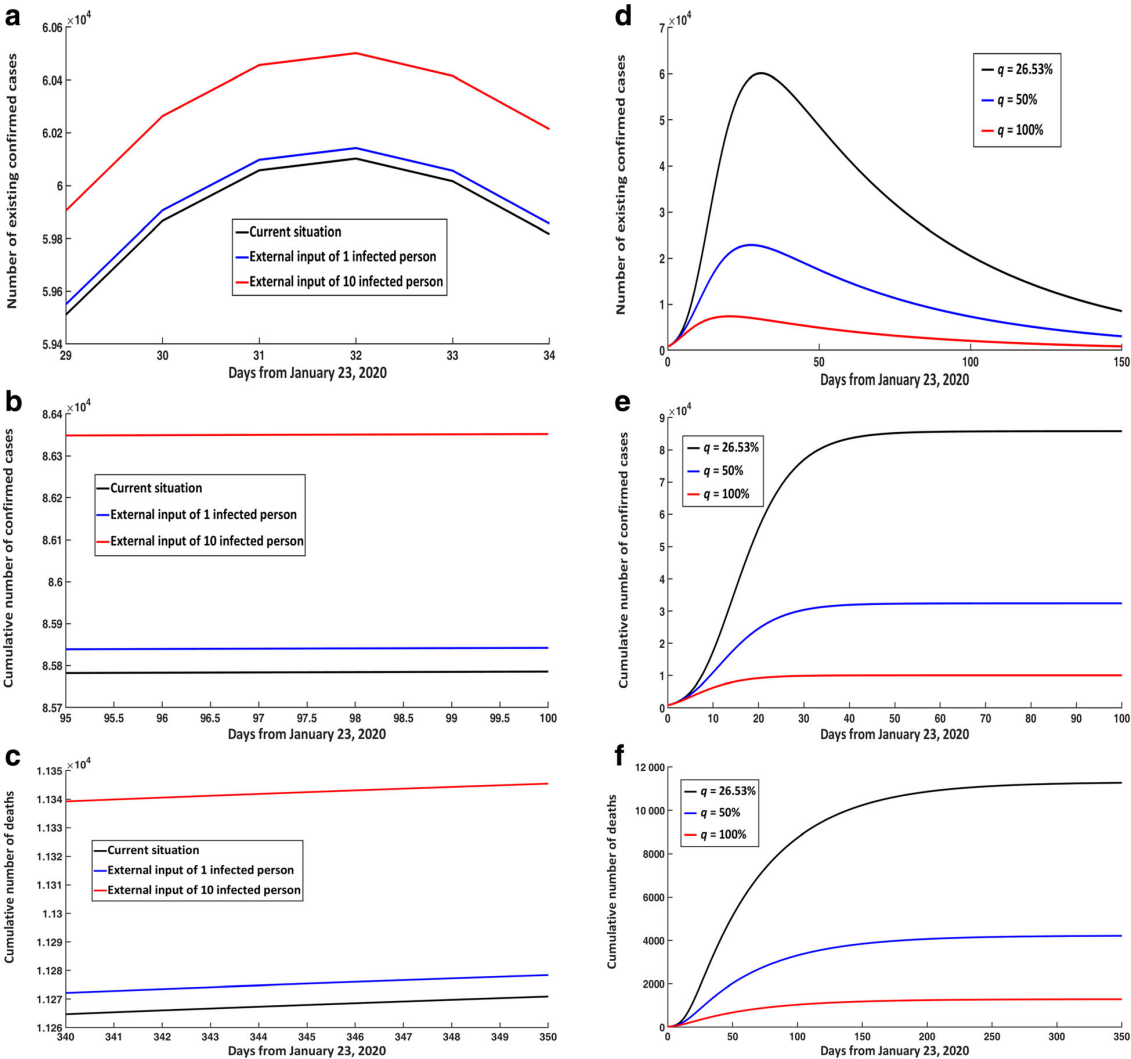
Impact of external input of infected persons and increasing qua rantine rate. Impact of external input of free infected persons on January 23rd on (**a**) existing and (**b**) cumulative confirmed cases, and (**c**) cumulative death numbers. Impact of increasing quarantine rate (*q*) on (**d**) existing and (**e**) cumulative confirmed cases, and (**f**) cumulative death numbers

If there was an external input of 1 or 10 free infected persons on January 23, 2020, then the number of existing confirmed cases at peak time compared with the current situation would increase by 40 and 400, respectively (Fig. 7a). The final cumulative number of confirmed cases would increase by 50 and 560, respectively (Fig. 7b), and the final cumulative number of deaths would increase by 10 and 80 cases, respectively (Fig. 7c).

In addition, if there was an exogenous input of 1 or 10 free infected persons every day since March 4, 2020, then the final cumulative number of confirmed cases compared with the current situation would increase by 50 and 610 cases, respectively (Figure S3D). The final cumulative number of deaths would increase by 40 and 410 cases, respectively (Figure S3E) (Supplementary p. 9–11).

Under the current situation, the quarantined rate of *q* was estimated to be 26.53%. If the quarantined rate of *q* was increased to 50%, then the number of existing confirmed cases at peak time would decrease by 37 220, equating to a decrease of 61.93%. Furthermore, the cumulative number of confirmed cases and deaths would decrease by 62.22% and 62.64%, respectively (Fig. 7d-f).

If the quarantined rate of *q* was increased by 100%, then the number of existing confirmed cases at peak time compared with the current situation would decrease by 52 718, equating to a decrease of 87.72%. The cumulative confirmed cases and deaths would decrease by 88.26% and 88.53%, respectively. Additionally, the peak time would be slightly ahead (Fig. 7d-f). Therefore, broad close-contact tracing played an important role in controlling COVID-19 transmission trends.

### COVID-19 epidemic trend in Hubei, China

The final cumulative number of confirmed cases in the Hubei Province would reach 72 023 (95% *CI:* 71 815–72 023). On January 23, 2020, the effective reproduction number of COVID-19 in the Hubei Province was 3.511 (95% *CI:* 3.489–3.534), and had dropped below 1.0 on February 7, 2020. The cumulative number of confirmed cases would begin to stabilize on April 1, 2020. Since March 13, 2020 the number of new confirmed cases would be less than 100 every day, and from April 1, 2020, newly confirmed cases would be less than 10 every day (Supplementary p. 15–20).

Similar to the entirety of the mainland of China, if isolation was relaxed compared with the current situation, then the number of existing confirmed cases would only increase by 3.36% by April 30, 2020. The cumulative number of confirmed cases and deaths would only increase by 0.02 and 0.17%, respectively. Based on the above analysis, we proposed a gradual relaxation of quarantine beginning on April 1st in the Hubei Province (Supplementary p. 20–22).

## Discussion

The emerging COVID-19 reported in Wuhan, Hubei Province, China has posed a great threat to public health and economic development since early 2020. An accurate estimate of the impact of strict preventive and control measures, as implemented by the Chinese government, on the COVID-19 epidemic trend was of great importance for preventing and controlling the spread of COVID-19 throughout China [20–23].

In the early stages of the COVID-19 epidemic, there was minimal literature on the COVID-19 transmission mechanism [16, 24]. To the best of our knowledge, this is an important and comprehensive report to predict the epidemic trend and transmission risk of SARS-CoV-2 infection after government intervention in the mainland of China.

In the current study, we developed a continuous SEIPQR model of COVID-19 transmission at the population level by considering the transmission mechanism of SARS-CoV-2 as well as the current prevention and control strategies, such as isolation and close-contact tracing as implemented by the Chinese government. The major difference among the published studies has been that models (1) and (2) comprehensively considered the role of various preventative and control measures, and distinguished the suspected cases compartment, which was more in line with the actual clinical diagnostic process. Based on reported data (seven different types of data) from the National Health Commission from January 23 to February 17, 2020, we estimated some initial values and important parameters by using the MCMC approach. Our study ultimately showed that the final cumulative number of confirmed cases in the mainland of China will be 86 763 (95% *CI:* 86067–87 460). We also predicted that the peak of existing confirmed cases would occur around February 23, 2020, with 60 890 cases (95% *CI:* 60350–61 431). Existing confirmed cases would slowly decline after the peak, which would approximately follow a chi-square distribution. Until March 15, 2020, the case fatality rate would increase to 6.42% (95% *CI:* 6.16–6.68%), and this estimated result may be slightly higher, mainly because we accounted for the number of deaths due to illness among infected patients that were not treated in isolation. Our study also shows that the effective reproduction number was 2.620 on January 23 and had dropped below 1.0 since February 5. More importantly, with intervention from the Chinese government, the total number of confirmed cases would be reduced by 99.85%.

Another concern from our study was the timing of relaxed isolation. After the relaxation of isolation, people’s social distance would become smaller, and factories and schools would gradually return to the pre-pandemic routines, so that the average contact rate (*c*) would increase. Through the model calculation, we found a critical level of contact rate: *c* = 4.356. If people did not pay attention to social distance after the isolation is relaxed, the average contact rate would be greater than this critical level; as such, relaxing isolation since February 24, 2020, would carry the risk of leading to a second peak of existing confirmed cases. However, if after the relaxation of isolation, people maintained a certain social distance, such that the average contact rate was less than this critical level, then extending isolation to March 16, 2020 or later would be expected to rapidly reduce the numbers of existing and cumulative confirmed cases, as well as that of fatality cases. Considering that the epidemic was relatively light outside of Hubei, it was feasible for provinces outside of Hubei to gradually relax isolation in early or middle March, according to the spread trends of COVID-19. On the other hand, extending isolation to April 1, 2020 or later in Hubei and Wuhan was necessary to comprehensively control COVID-19 spread. These results had important implications on the government’s public health decisions that would promote maximal epidemic control with minimal impact on both people’s lives and the economy.

Furthermore, we evaluated the impact of isolation intensity, delayed diagnosis, external input of free infected persons, and increased coverage of follow-up on close-contacts on the COVID-19 epidemic trend in the mainland of China. If coverage of close-contact tracing was increased to 100%, the cumulative number of confirmed and death cases would decrease by 88.26 and 88.53%, respectively, and the peak time would be earlier. On the contrary, the total number of confirmed and death cases would increase by 8.72 and 9.44%, respectively, with a 1-day delay in diagnosis in unquarantined infected patients after January 23, 2020, corresponding to the delay of the peak time. These results once again emphasized the validity of government decision-making as well as the importance of early diagnosis and extensive medical isolation. Additionally, our study showed that external input of a single infected person from January 23, 2020 would lead to an increase of 50 cumulative confirmed cases and 10 cumulative deaths. Therefore, the strict immigrant monitoring measures that were implemented in different regions were critical.

In addition, according to our model’s calculation, the number of infected patients who failed to seek medical treatment in a timely manner could exceed 10 000 (Supplementary p. 8–9). Had they been treated in time, the number of confirmed cases and deaths would have been greatly reduced.

There were some limitations to this study that must be considered. First, we ignored the effect of uneven population distribution and assumed that the total population was homogeneously distributed. Second, we ignored the differences in individual susceptibility and we assumed that infection susceptibility for all individuals in the free environment was the same; whereas, in actuality, adults and older people are more likely to be infected by SARS-CoV-2. Third, we did not take into account the limitation of medical resources, such as health care workers and medical protective equipment. Fourth, the effects of viral variation on infectivity and virulence, which might be directly related to the number of confirmed cases and deaths, were not taken into account. Finally, our model forecasts are based on reported data from the National Health Commission of China before February 17, 2020. Therefore, it is possible that treatment options such as Remdesivir’s approval for clinical use, the use of serum in convalescent patients, and mesenchymal stem cells may significantly reduce mortality from COVID-19 [25–28], which would lead to an overestimation of mortality in our study. Furthermore, since the study was constructed from reported data and some parameters were calculated based on preliminary studies, these data came from heterogeneous sources, which may have introduced biases. It was important to note that when we predicted the epidemic trend of COVID-19 without any control measures, due to the small amount of reported data, the estimated parameters might have certain errors and the predicted results might represent an over-prediction.

## Conclusions

Based on real reported data and a SEIPQR model of SARS-CoV-2 transmission, the pneumonia outbreak caused by SARS-CoV-2 in China had been effectively controlled. The series of quarantine measures adopted by the Chinese government since January 23, 2020 were necessary and effective. Postponing the relaxation of isolation until March 16, 2020 or later decreased the existing confirmed cases and case fatality rates. April 1, 2020 was supposed to be a reasonable date to relax the isolation in Hubei and Wuhan. Early detection and patient isolation can effectively reduce the scale of infection and mortality. Measures such as broad close-contact tracing and strict monitoring of infected persons entering the country were also necessary and had proven to be very effective. These results provided a quantitative reference for government agencies in China as well as in other countries and regions to prevent and control COVID-19.

## Supplementary information

**Additional file 1.**

## Data Availability

Not applicable.

## Abbreviations

COVID-19: Corona virus disease 2019
WHO: World Health Organization
SARS-CoV-2: Severe acute respiratory syndrome coronavirus 2
*CI*: Confidence interval
MCMC: Markov Chain Monte Carlo
SEIPQR: Susceptible-exposed-infectious-suspected-confirmed-recovered
SEIQR: Susceptible-exposed-infectious-confirmed-recovered

## References

[1] Special Expert Group for Control of the Epidemic of Novel Coronavirus Pneumonia of the Chinese Preventive Medicine Association (2020). An update on the epidemiological characteristics of novel coronavirus pneumonia (COVID-19). Zhonghua Liu Xing Bing Xue Za Zhi.

[2] Backer JA, Klinkenberg D, Wallinga J (2020). Incubation period of 2019 novel coronavirus (2019-nCoV) infections among travellers from Wuhan, China, 20-28 January 2020. Euro Surveill..

[3] Huang C, Wang Y, Li X, Ren L, Zhao J, Hu Y (2020). Clinical features of patients infected with 2019 novel coronavirus in Wuhan, China. Lancet.

[4] Lu R, Zhao X, Li J, Niu P, Yang B, Wu H (2020). Genomic characterisation and epidemiology of 2019 novel coronavirus: implications for virus origins and receptor binding. Lancet..

[5] Chan JFW, Yuan S, Kok KH, To KK, Chu H, Yang J (2020). A familial cluster of pneumonia associated with the 2019 novel coronavirus indicating person-to-person transmission: a study of a family cluster. Lancet.

[6] Jiang S, Du L, Shi Z (2020). An emerging coronavirus causing pneumonia outbreak in Wuhan, China: calling for developing therapeutic and prophylactic strategies. Emerg Microbes Infect.

[7] National Health Commission of the People’s Republic of China. Daily report on COVID-19. http://www.nhc.gov.cn/xcs/yqtb/list_gzbd.shtml. Accessed 19 Feb 2020. 10.46234/ccdcw2020.082 PMC8392946 34594648

[8] Wang C, Horby PW, Hayden FG, Gao GF (2020). A novel coronavirus outbreak of global health concern. Lancet..

[9] Li Q, Guan X, Wu P, Wang X, Zhou L, Tong Y, et al. Early transmission dynamics in Wuhan, China, of novel coronavirus-infected pneumonia. N Engl J Med. 2020;382:1199–207. 10.1056/NEJMoa2001316 PMC7121484 31995857

[10] Zhao S, Lin Q, Ran J, Musa S, Yang G, Wang W (2020). Preliminary estimation of the basic reproduction number of novel coronavirus (2019-nCoV) in China, from 2019 to 2020: a data-driven analysis in the early phase of the outbreak. Int J Infect Dis.

[11] Al-qaness MAA, Ewees AA, Fan H, Aziz MAE (2020). Optimization method for forecasting confirmed cases of COVID-19 in China. J Clin Med.

[12] Chen T, Rui J, Wang Q, Zhao ZY, Cui JA, Yin L (2020). A mathematical model for simulating the phase-based transmissibility of a novel coronavirus. Infect Dis Poverty.

[13] Dye C, Gay N (2003). Modeling the SARS epidemic. Science.

[14] Chen N, Zhou M, Dong X, Qu J, Gong F, Han Y (2020). Epidemiological and clinical characteristics of 99 cases of 2019 novel coronavirus pneumonia in Wuhan, China: a descriptive study. Lancet.

[15] Althaus CL. Estimating the reproduction number of Ebola virus (EBOV) during the 2014 outbreak in West Africa. PLoS Curr. 2014;6. 10.1371/currents.outbreaks.91afb5e0f279e7f29e7056095255b288. 10.1371/currents.outbreaks.91afb5e0f279e7f29e7056095255b288 PMC4169395 25642364

[16] Tang B, Wang X, Li Q, Bragazzi NL, Tang S, Xiao Y (2020). Estimation of the transmission risk of the 2019-nCoV and its implication for public health interventions. J Clin Med.

[17] Park SW, Champredon D, Earn DJD, Li M, Weitz JS, Grenfell BT, et al. Reconciling early-outbreak preliminary estimates of the basic reproductive number and its uncertainty: a new framework and applications to the novel coronavirus (2019-nCoV) outbreak. medRxiv. 2020. 10.1101/2020.01.30.20019877. 10.1098/rsif.2020.0144 PMC7423425 32693748

[18] Chernozhukov V, Hong H (2003). An MCMC approach to classical estimation. J Econom.

[19] Cauchemez S, Carrat F, Viboud C, Valleron AJ, Boëlle PY (2004). A Bayesian MCMC approach to study transmission of influenza: application to household longitudinal data. Stat Med.

[20] Zhang J, Lou J, Ma Z (2005). Wu Jl. A compartmental model for the analysis of SARS transmission patterns and outbreak control measures in China. Appl Math Comput.

[21] Quilty BJ, Clifford S, Flasche S, Eggo RM (2020). Effectiveness of airport screening at detecting travellers infected with novel coronavirus (2019-nCoV). Euro Surveill.

[22] Bassetti M, Vena A, Roberto GD (2020). The novel Chinese coronavirus (2019-nCoV) infections: challenges for fighting the storm. Eur J Clin Investig.

[23] Wang FS, Zhang C (2020). What to do next to control the 2019-nCoV epidemic?. Lancet..

[24] Gilbert M, Pullano G, Pinotti F, Valdano E, Poletto C, Boëlle PY (2020). Preparedness and vulnerability of African countries against importations of COVID-19: a modelling study. Lancet..

[25] Wang M, Cao R, Zhang L, Yang X, Liu J, Xu M (2020). Remdesivir and chloroquine effectively inhibit the recently emerged novel coronavirus (2019-nCoV) in vitro. Cell Res.

[26] Chen L, Xiong J, Bao L, Shi Y (2020). Convalescent plasma as a potential therapy for COVID-19. Lancet Infect Dis.

[27] Matthay MA, Calfee CS, Zhuo H, Thompson BT, Wilson JG, Levitt JE (2019). Treatment with allogeneic mesenchymal stromal cells for moderate to severe acute respiratory distress syndrome (START study): a randomised phase 2a safety trial. Lancet Respir Med.

[28] Ji F, Li L, Li Z, Jin Y, Liu W. Mesenchymal stem cells as a potential treatment for critically ill patients with coronavirus disease 2019. Stem Cells Transl Med. 2020;9:813–4. 10.1002/sctm.20-0083. 10.1002/sctm.20-0083 PMC7264790 32320535

