# Supplementary material for: Transmission patterns of COVID-19 in the mainland of China and the efficacy of different control strategies: a data- and model-driven study
Source: Infect Dis Poverty. 2020 Jul 6;9:83. doi: 10.1186/s40249-020-00709-z (PMC7338105; doi:10.1186/s40249-020-00709-z)
Supplement: Supplementary file 1 — Additional file 1. [file 40249_2020_709_MOESM1_ESM.docx]

**Transmission patterns of COVID-19 in mainland China and the efficacy of different control strategies: a data- and model-driven study**

Jian Zu, PhD^1, *^, Miaolei Li, MD^1^, Zongfang Li, MD, PhD ^2,3^, Mingwang Shen, PhD^4^, Yanni Xiao,PhD ^1^, Fanpu Ji, MD, PhD^2,3,5 *^

^1^ School of Mathematics and Statistics, Xi’an Jiaotong University, Xi’an, Shaanxi, 710049, P. R. China

^2^ National & Local Joint Engineering Research Center of Biodiagnosis and Biotherapy, the Second Affiliated Hospital, Xi’an Jiaotong University, Xi’an, China

^3^ Key Laboratory of Environment and Genes Related to Diseases, Xi'an Jiaotong University, Ministry of Education of China, Xi'an, China

^4^ School of Public Health, Health Science Center, Xi’an Jiaotong University, Xi’an, Shaanxi, 710061, P. R. China

^5^ Department of Infectious Diseases, the Second Affiliated Hospital of Xi’an Jiaotong University, Xi’an, China

***Correspondence: Jian Zu**, School of Mathematics and Statistics, Xi’an Jiaotong University, Xi’an, Shaanxi, 710049, P. R. China (Email address: [jianzu@xjtu.edu.cn](mailto:jianzu@xjtu.edu.cn)). **Fanpu Ji**, Department of Infectious Diseases, the Second Affiliated Hospital of Xi’an Jiaotong University, Xi’an, China, 157 Xi Wu Road, Xi'an 710004, Shaanxi Province, PR. China. (Email address:[jifanpu1979@163.com](mailto:jifanpu1979@163.com) or infection@xjtu.edu.cn).

**Supplementary material**

**S.1 Daily reported data used in this article**

The data in Table S1 were collected from the website of National Health Commission of China, which were used to estimate the parameters and initial values of models (1) and (2) in the mainland of China.

**Table S1.** Reported data for COVID-19 in the mainland of China from Jan 23 to Feb 17, 2020.^1-3^

| Date | Cumulative confirmed cases | Existing suspected cases | Cumulative deaths | Cumulative recovered cases | Existing medical observations | Cumulative suspected cases | Existing confirmed cases |
| --- | --- | --- | --- | --- | --- | --- | --- |
| 2020/1/23 | 830 | -- | 25 | 34 | 8420 | 1072 | 771 |
| 2020/1/24 | 1287 | 1965 | 41 | 38 | 13967 | 2190 | 1208 |
| 2020/1/25 | 1975 | 2684 | 56 | 49 | 21556 | 3499 | 1870 |
| 2020/1/26 | 2744 | 5794 | 80 | 51 | 30453 | 7305 | 2613 |
| 2020/1/27 | 4515 | 6973 | 106 | 60 | 44132 | 9382 | 4349 |
| 2020/1/28 | 5974 | 9239 | 132 | 103 | 59990 | 12630 | 5739 |
| 2020/1/29 | 7711 | 12167 | 170 | 124 | 81947 | 16778 | 7417 |
| 2020/1/30 | 9692 | 15238 | 213 | 171 | 102427 | 21590 | 9308 |
| 2020/1/31 | 11791 | 17988 | 259 | 243 | 118478 | 26609 | 11289 |
| 2020/2/1 | 14380 | 19544 | 304 | 328 | 137594 | 31171 | 13748 |
| 2020/2/2 | 17205 | 21558 | 361 | 475 | 152700 | 36344 | 16369 |
| 2020/2/3 | 20438 | 23214 | 425 | 632 | 171329 | 41416 | 19381 |
| 2020/2/4 | 24324 | 23260 | 490 | 892 | 185555 | 45387 | 22942 |
| 2020/2/5 | 28018 | 24702 | 563 | 1153 | 186354 | 50715 | 26302 |
| 2020/2/6 | 31161 | 26359 | 636 | 1540 | 186045 | 55548 | 28985 |
| 2020/2/7 | 34546 | 27657 | 722 | 2050 | 189660 | 59762 | 31774 |
| 2020/2/8 | 37198 | 28942 | 811 | 2649 | 188183 | 63678 | 33738 |
| 2020/2/9 | 40171 | 23589 | 908 | 3281 | 187518 | 67686 | 35982 |
| 2020/2/10 | 42638 | 21675 | 1016 | 3996 | 187728 | 71222 | 37626 |
| 2020/2/11 | 44653 | 16067 | 1113 | 4740 | 185037 | 74564 | 38800 |
| 2020/2/12 | 59804 | 13435 | 1367 | 5911 | 181386 | 77371 | 52526 |
| 2020/2/13 | 63851 | 10109 | 1380 | 6723 | 177984 | 79821 | 55748 |
| 2020/2/14 | 66492 | 8969 | 1523 | 8096 | 169039 | 82098 | 56873 |
| 2020/2/15 | 68500 | 8228 | 1665 | 9419 | 158764 | 84016 | 57416 |
| 2020/2/16 | 70548 | 7264 | 1770 | 10844 | 150539 | 85579 | 57934 |
| 2020/2/17 | 72436 | 6242 | 1868 | 12552 | 141552 | 87011 | 58016 |

The data in Table S2 were collected from the website of National Health Commission of China, which were used to estimate the parameters and initial values of models (S1) and (S2).

**Table S2.** Reported data for COVID-19 in the mainland of China from Jan 10 to Jan 22, 2020.^1-3^

| Date | Cumulative  confirmed cases | Cumulative deaths | Cumulative recovered cases |
| --- | --- | --- | --- |
| 2020/1/10 | 41 | 1 | 0 |
| 2020/1/11 | 41 | 1 | 0 |
| 2020/1/12 | 41 | 1 | 0 |
| 2020/1/13 | 41 | 1 | 0 |
| 2020/1/14 | 41 | 1 | 0 |
| 2020/1/15 | 41 | 2 | 5 |
| 2020/1/16 | 45 | 2 | 8 |
| 2020/1/17 | 62 | 2 | 12 |
| 2020/1/18 | 198 | 3 | 17 |
| 2020/1/19 | 275 | 4 | 18 |
| 2020/1/20 | 291 | 6 | 25 |
| 2020/1/21 | 440 | 9 | 25 |
| 2020/1/22 | 571 | 17 | 25 |

The data in Table S3 were collected from the website of National Health Commission of China and Health Commission of Hubei Province, which were used to estimate the parameters and initial values of models (1) and (2) in Hubei Province.

**Table S3.** Reported data for COVID-19 in Hubei Province from Jan 23 to Feb 17, 2020.^1-3^

| Date | Cumulative confirmed cases | Existing confirmed cases | Cumulative deaths | Cumulative recovered cases | Existing medical observations |
| --- | --- | --- | --- | --- | --- |
| 2020/1/23 | 549 | 494 | 24 | 31 | 2776 |
| 2020/1/24 | 729 | 658 | 39 | 32 | 4711 |
| 2020/1/25 | 1052 | 958 | 52 | 42 | 6904 |
| 2020/1/26 | 1423 | 1303 | 76 | 44 | 9103 |
| 2020/1/27 | 2714 | 2567 | 100 | 47 | 15559 |
| 2020/1/28 | 3554 | 3349 | 125 | 80 | 20360 |
| 2020/1/29 | 4586 | 4334 | 162 | 90 | 26632 |
| 2020/1/30 | 5806 | 5486 | 204 | 116 | 32340 |
| 2020/1/31 | 7153 | 6738 | 249 | 166 | 36838 |
| 2020/2/1 | 9074 | 8565 | 294 | 215 | 43121 |
| 2020/2/2 | 11177 | 10532 | 350 | 295 | 48171 |
| 2020/2/3 | 13522 | 12712 | 414 | 396 | 58544 |
| 2020/2/4 | 16678 | 15679 | 479 | 520 | 66764 |
| 2020/2/5 | 19665 | 18473 | 549 | 633 | 64127 |
| 2020/2/6 | 22112 | 20677 | 618 | 817 | 64057 |
| 2020/2/7 | 24953 | 23139 | 699 | 1115 | 67802 |
| 2020/2/8 | 27100 | 24881 | 780 | 1439 | 70438 |
| 2020/2/9 | 29631 | 26965 | 871 | 1795 | 73127 |
| 2020/2/10 | 31728 | 28532 | 974 | 2222 | 76207 |
| 2020/2/11 | 33366 | 29659 | 1068 | 2639 | 77195 |
| 2020/2/12 | 48206 | 43455 | 1310 | 3441 | 77308 |
| 2020/2/13 | 51986 | 46537 | 1318 | 4131 | 77685 |
| 2020/2/14 | 54406 | 48175 | 1457 | 4774 | 77323 |
| 2020/2/15 | 56249 | 49030 | 1596 | 5623 | 74261 |
| 2020/2/16 | 58182 | 49847 | 1696 | 6639 | 71613 |
| 2020/2/17 | 59989 | 50338 | 1789 | 7862 | 69270 |

**S.2 Comparison with the daily reported data**

The comparison between the estimated values by models (1) and (2) and the actual reported data was shown in Figure S1. We compared the model estimation results with 7 different types of reported data in Table S1. It can be seen that the estimated results of the model fitted well with the actual reported data, the root mean square error (RMSE) was 2096. This comparison suggested that the estimated values by model were very good agreement with real reported data, serving as a validation of the model. Therefore, models (1) and (2) and the estimated parameter values can be used to predict the future development trend of COVID-19 in the mainland of China.

(A)


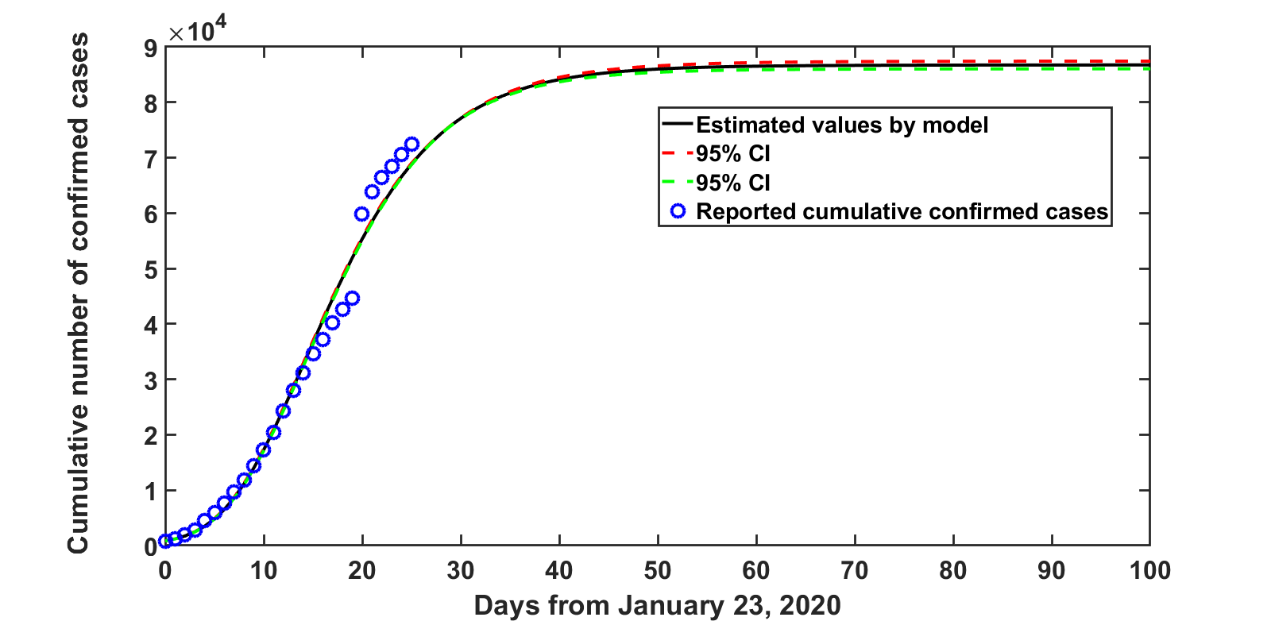


(B)


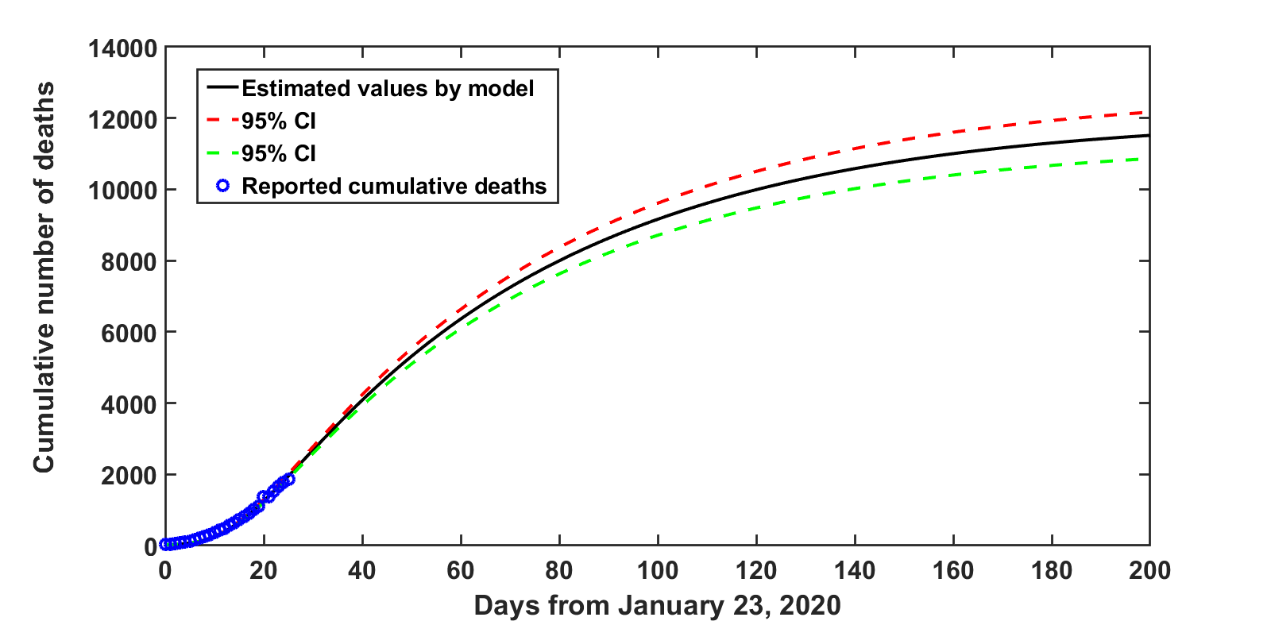


(C)


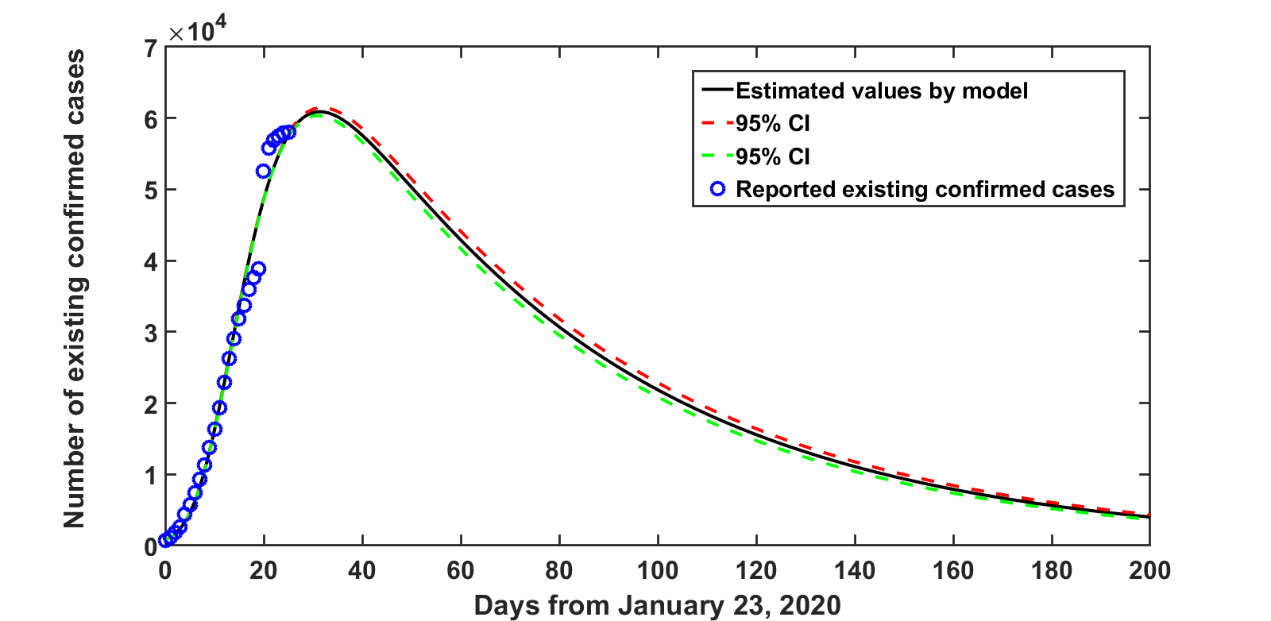


(D)


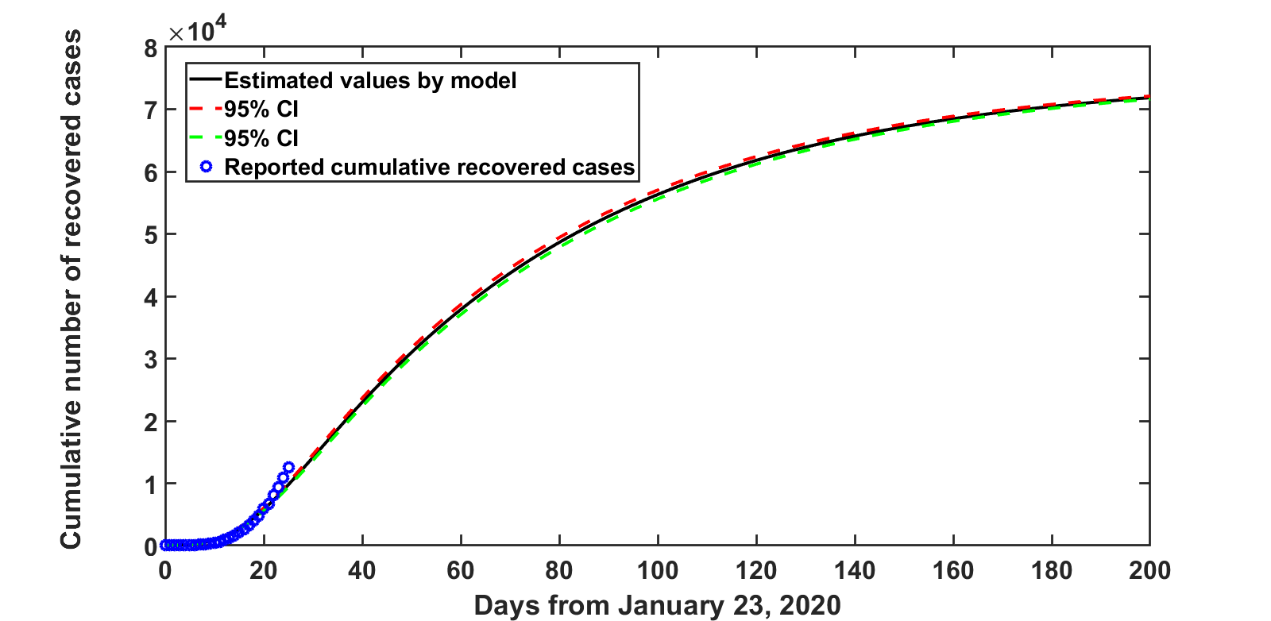


(E)


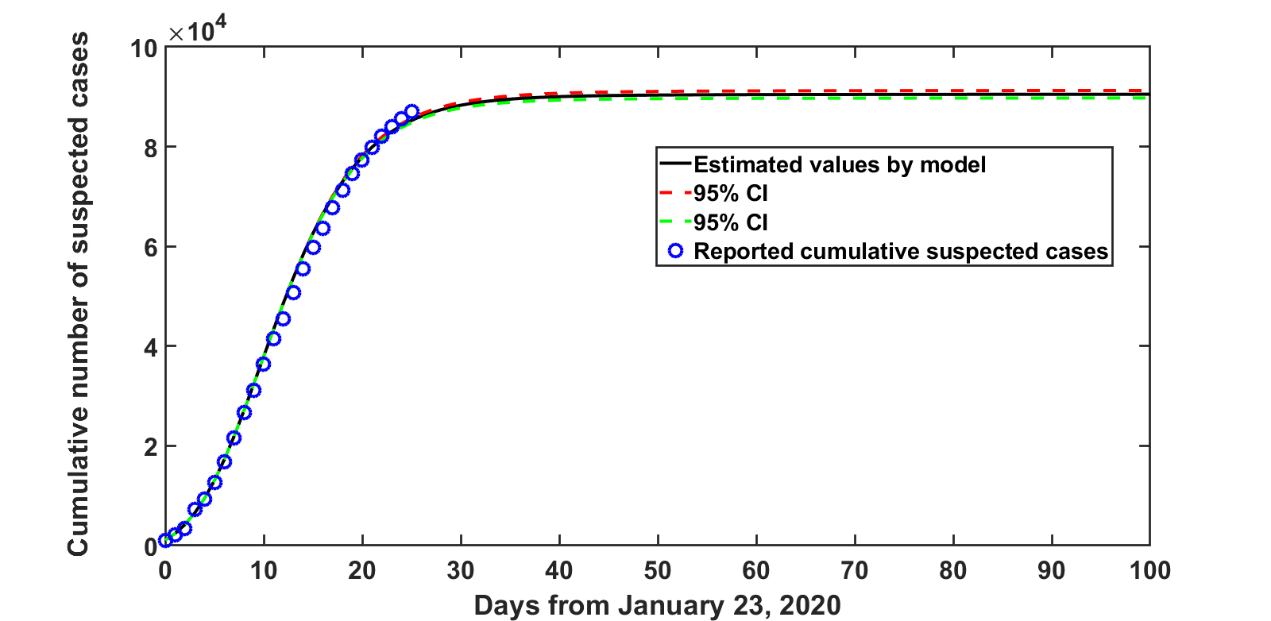


(F)


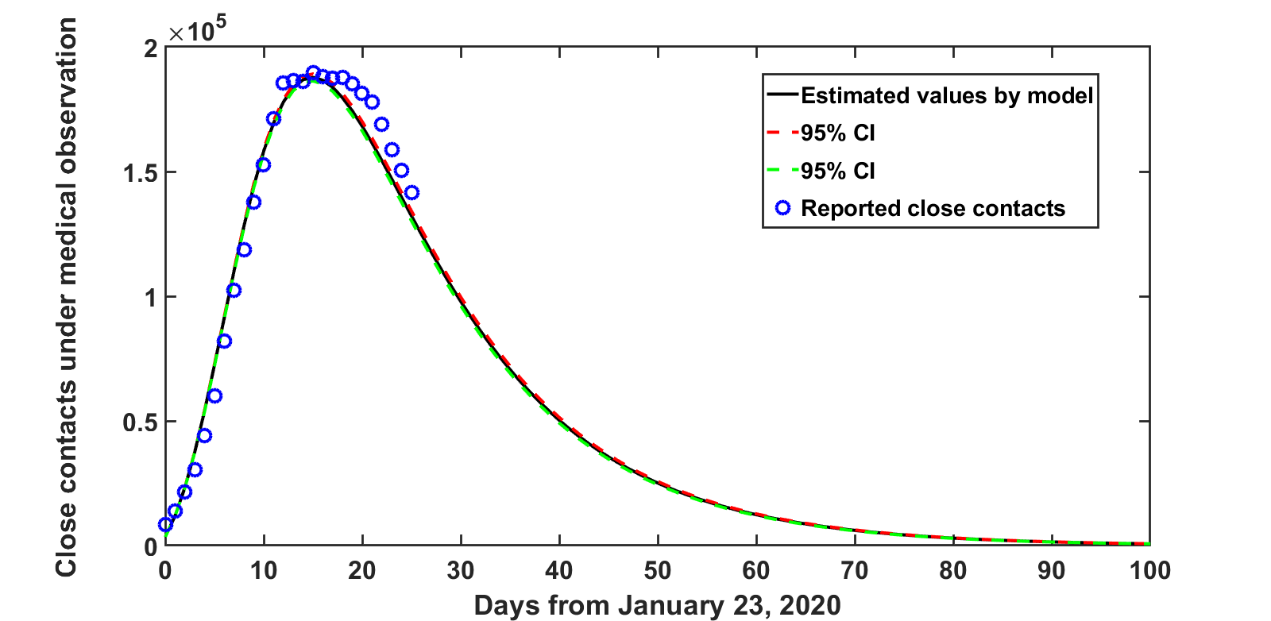


(G)


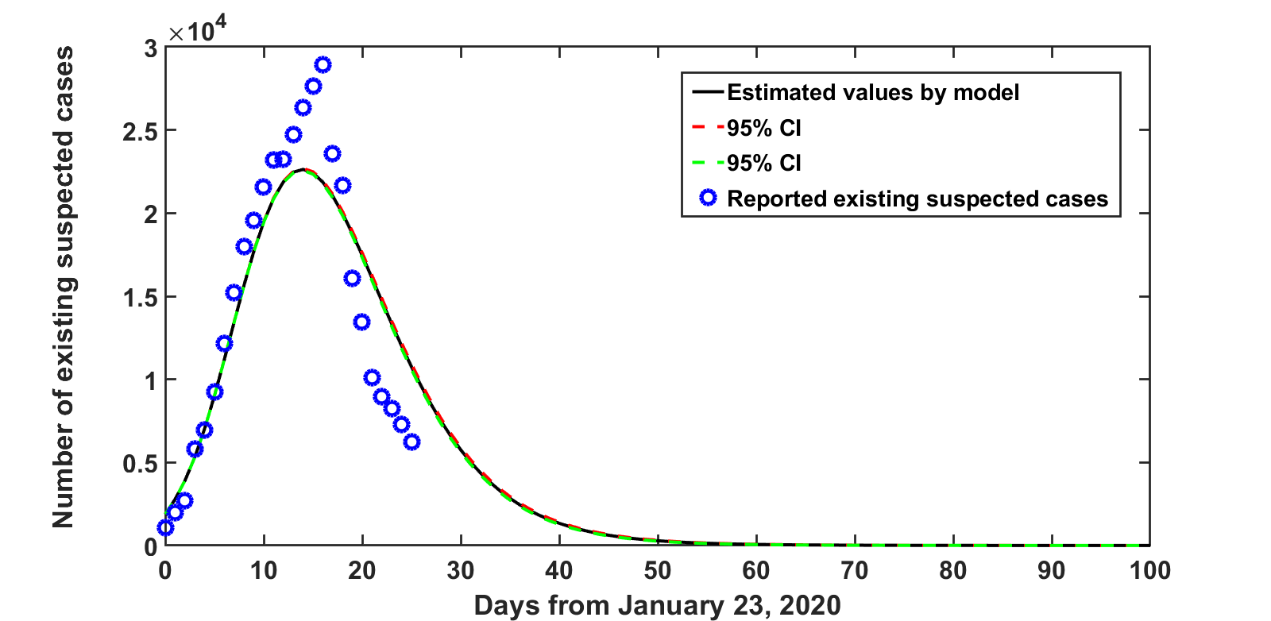


**Figure S1.** Comparison of estimated values by model with real reported data. (A) Cumulative number of confirmed cases over time. (B) Cumulative number of deaths over time. (C) Number of existing confirmed cases over time. (D) Cumulative number of recovered cases over time. (E) Cumulative number of suspected cases over time. (F) Close contacts under medical observation over time. (G) Number of existing suspected cases over time. The red dashed line was the upper limit of the 95% CI, and the green dashed line was the lower limit of the 95% CI.

**S.3 Epidemic trend of free infected and latent people**

By calculating the model (1) using the estimated parameters and initial values in Table 1, we obtained the epidemic trend of free infected and latent people over time (Figure S2). From Figure S2, we can see that the number of free infected people reached to its peak around Feb 7, 2020, with a peak of 12,001 cases. The number of free latent people reached to its peak around Feb 1, 2020, with 11,368 cases. The number of traced latent people reached to its peak around Feb 2, 2020, with 6,301 cases.

(A)


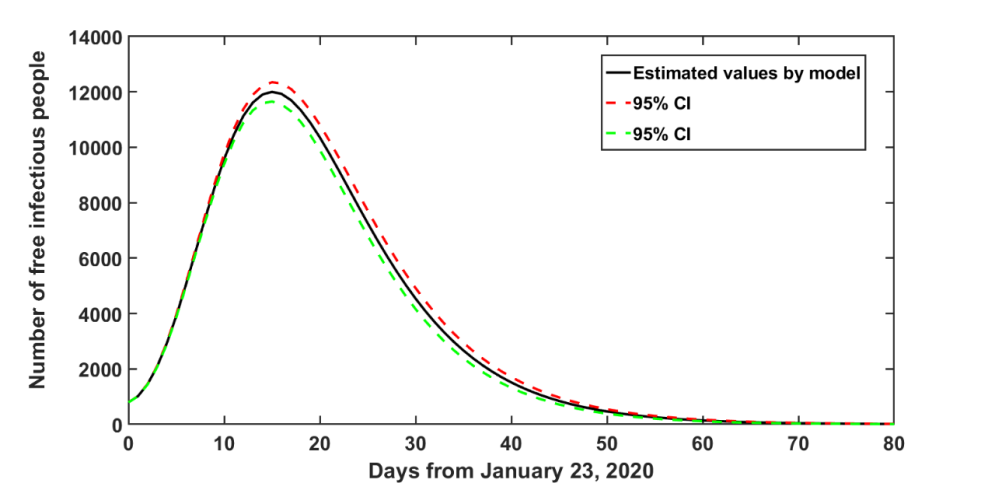


(B)


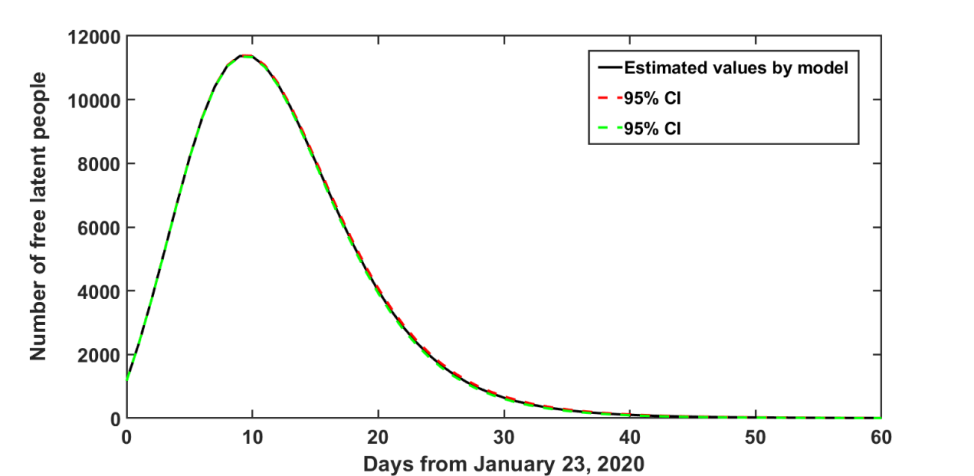


(C)


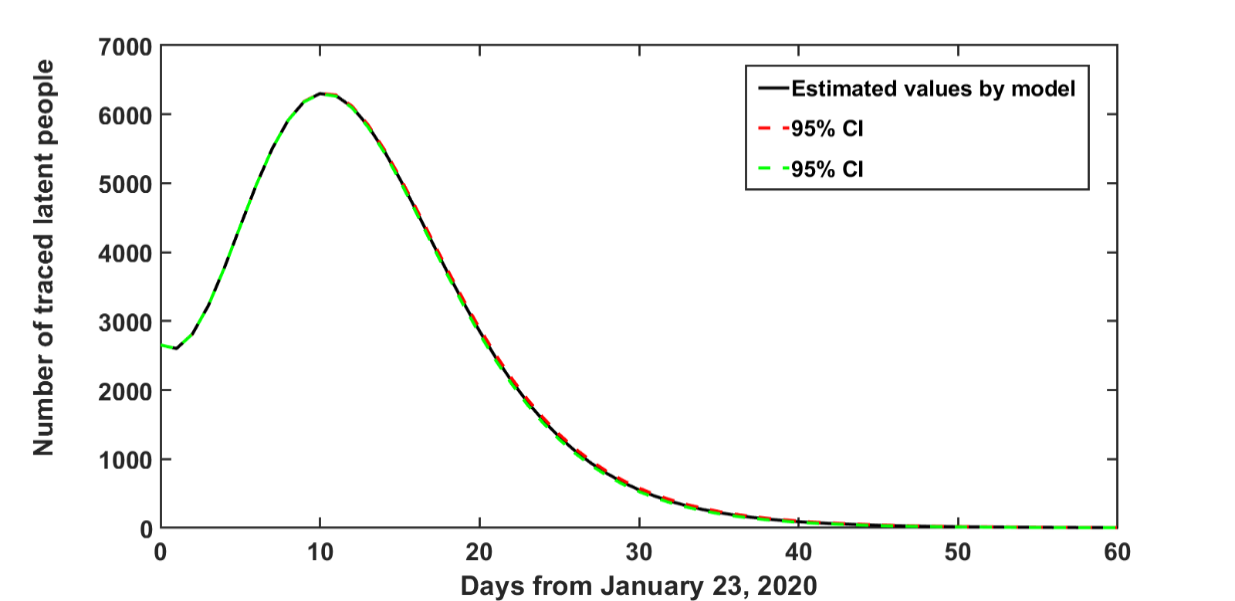


**Figure S2.** Epidemic trend of free infected and latent people. (A) Number of free infected people over time. (B) Number of free latent people over time. (C) Number of traced latent people over time. The red dashed line was the upper limit of the 95% CI, and the green dashed line was the lower limit of the 95% CI.

**S.4 Impact of external input of free infected persons**

By direct calculation, we find that if there was an external input of 1 or 10 free infected person on Jan 23, 2020, then compared with the current situation, at the peak time, the number of existing confirmed cases would increase by 40 and 400, respectively (Figure S3A); the finally cumulative number of confirmed cases would increase by 50 and 560, respectively (Figure S3B); the finally cumulative number of deaths would increase by 10 and 80 cases, respectively (Figure S3C).

In addition, from Mar 4, there have been confirmed cases imported from outside China every day. If since Mar 4, there was an external input of 1 or 10 free infected person every day, then compared with the current situation, the finally cumulative number of confirmed cases would increase by 50 and 610 cases, respectively (Figure S3D); the finally cumulative number of deaths would increase by 40 and 410 cases, respectively (Figure S3E). In particular, with the gradual relaxation of quarantine measures, the impact of imported cases will be more severe. Therefore, it is very important to strengthen the strict detection and isolation of overseas cases.

(A)


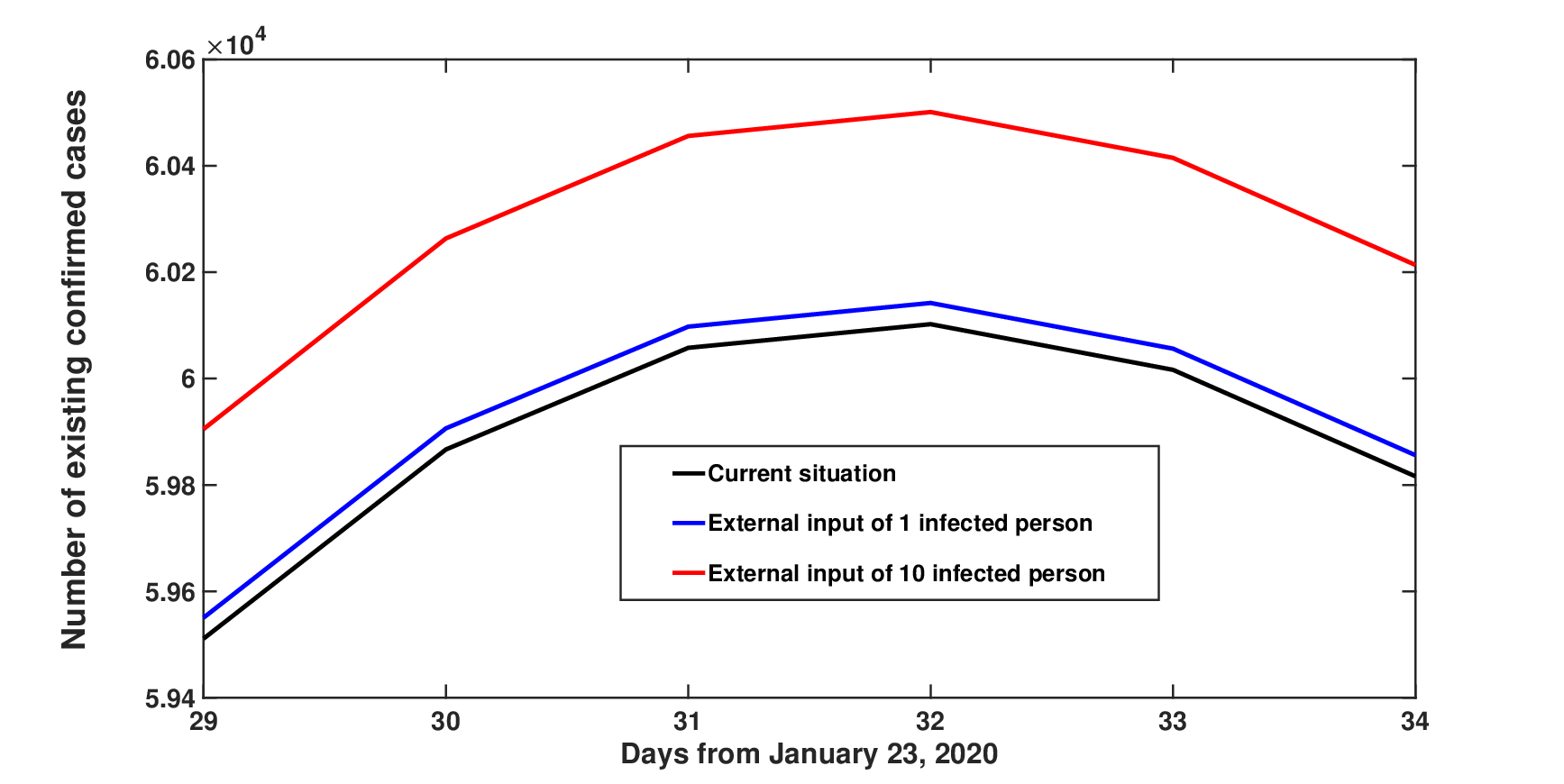


(B)


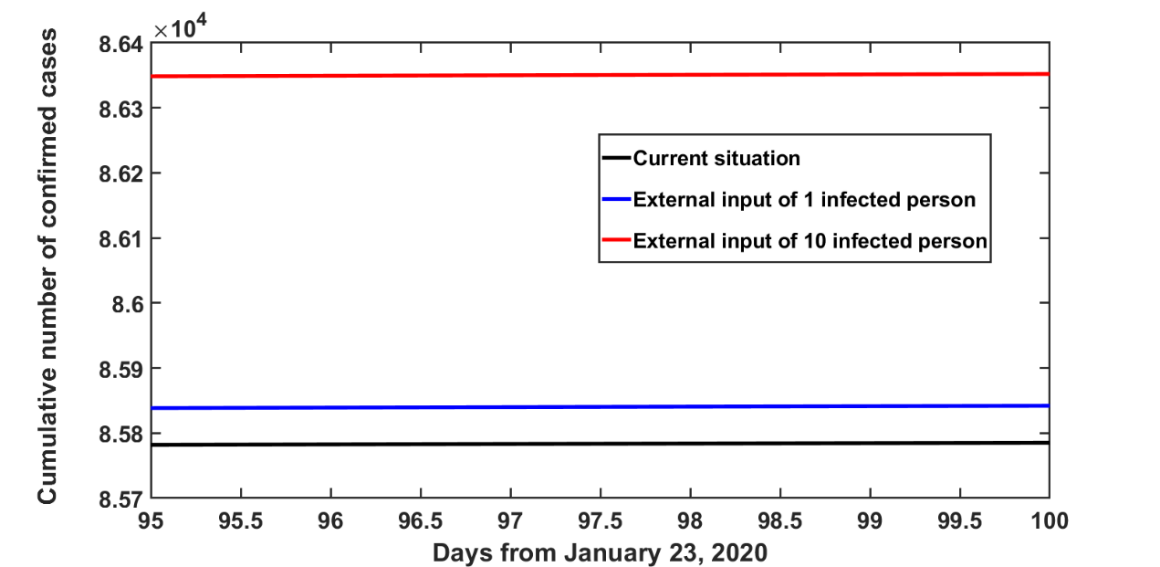


(C)


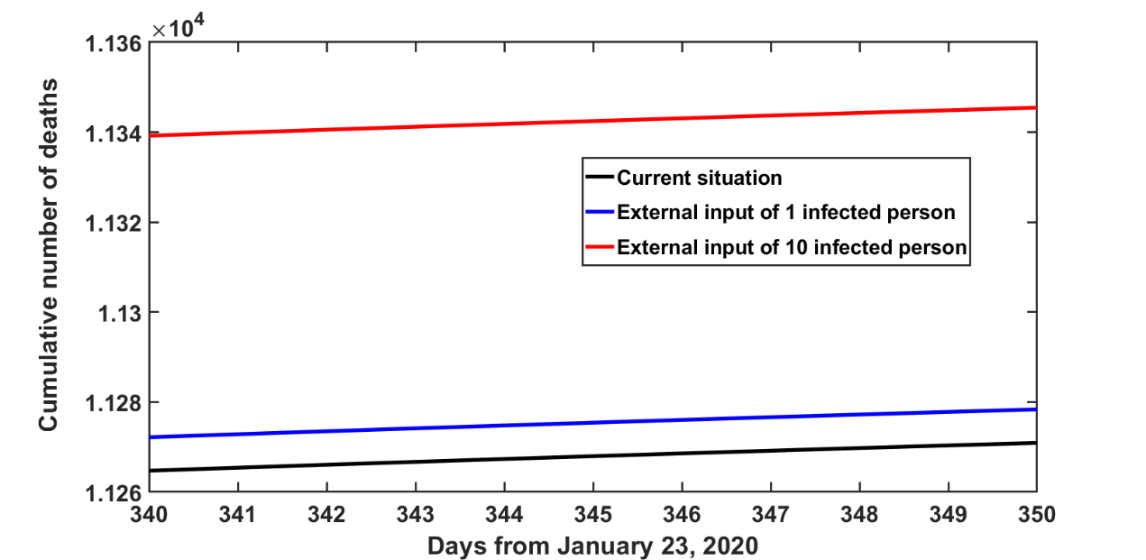


(D)

**
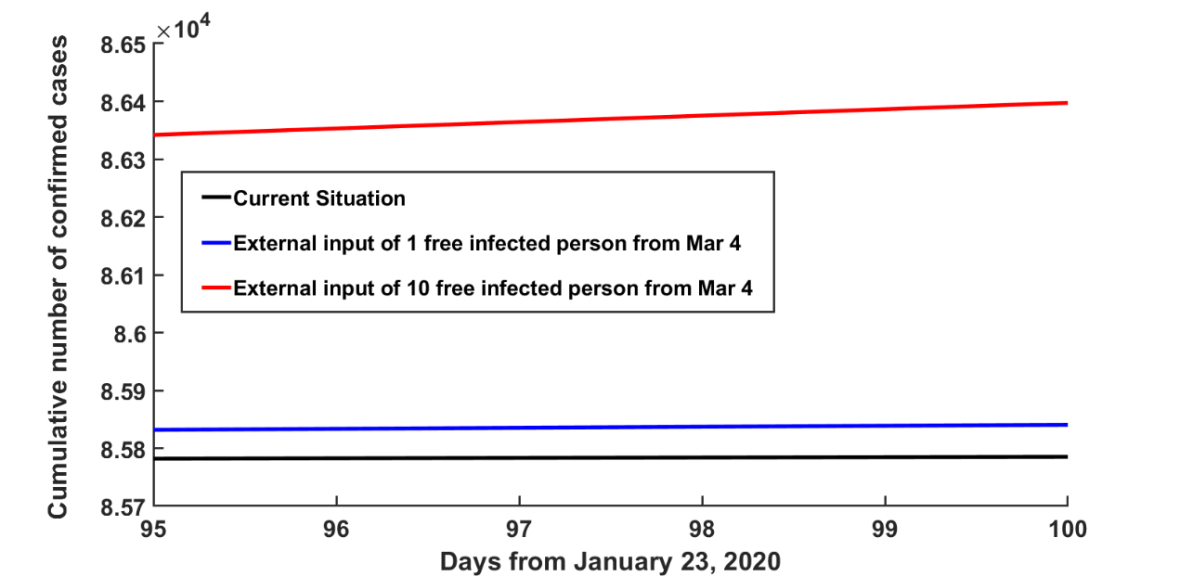
**

(E)

**
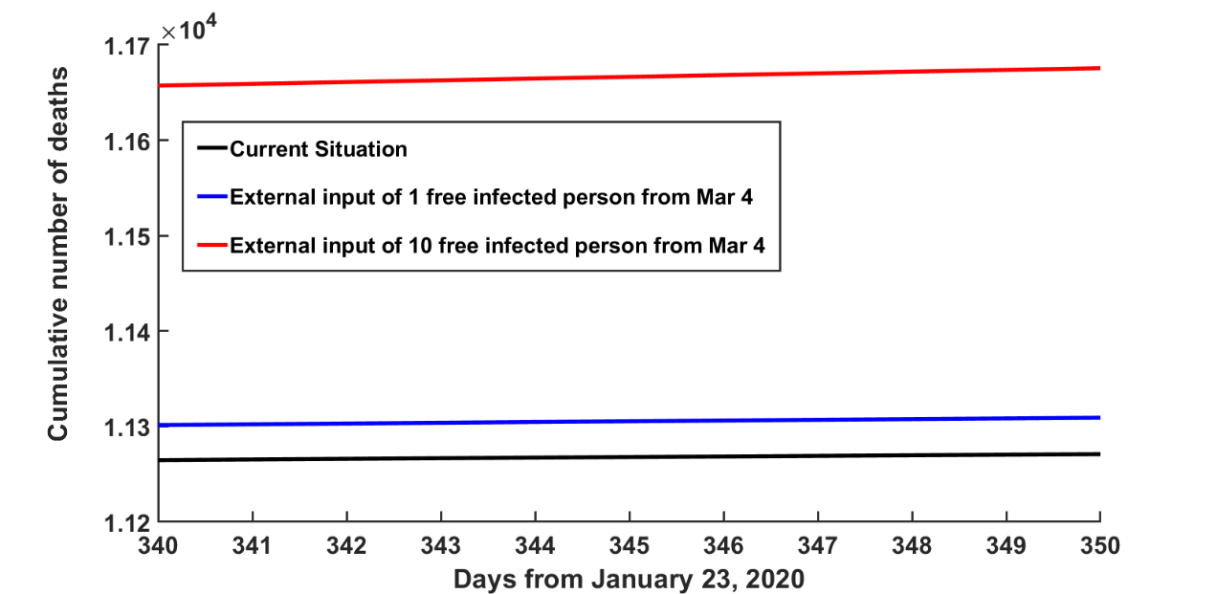
**

**Figure S3.** Impact of external input of free infected persons. (A) Impact of external input of free infected person on Jan 23 on the daily number of existing confirmed cases. (B) Impact of external input of free infected person on Jan 23 on the cumulative number of confirmed cases. (C) Impact of external input of free infected person on Jan 23 on the cumulative number of deaths. (D) Impact of external input of free infected person from Mar 4 on the cumulative number of confirmed cases. (E) Impact of external input of free infected person from Mar 4 on the cumulative number of deaths.

**S.5 Epidemic trend of COVID-19 without any control measures**

When we did not take any preventive and control measures before Jan 23, we reduced 3 compartments in model (1) and obtained an SEIQR model (S1):

$\begin{matrix} dS/dt=-\frac{\beta c(t)S}{N(t)}(kE+I), \\ dE/dt=\frac{\beta c(t)S}{N(t)}(kE+I)-\varepsilon E, \\ dI/dt=\varepsilon E-d_{iq}I-\delta I, \\ dQ/dt=d_{iq}I-\delta Q-\gamma Q, \\ dR/dt=\gamma Q. \end{matrix}$ (S1)

Here, $N(t)=S(t)+E(t)+I(t)+Q(t)+R(t)$represented the total size of the population. The cumulative number of confirmed cases$H$and cumulative number of deaths$Z$were described by (S2):

$\begin{matrix} dH/dt=d_{iq}I, \\ dZ/dt=\delta I+\delta Q. \end{matrix}$ (S2)

If there were no prevention and control measures, then the possibility of person-to-person contact during the China Spring Festival might be greater, other times might be relatively less, so we assumed that the contact rate obeyed the following normal distribution:

$c(t)=c_{0}\exp(-\frac{(t-\mu)^{2}}{2\sigma^{2}})$ (S3)

Using the method of next-generation matrix, we obtained the effective reproductive number of model (S1), which was given by:

$R(t)=\left[\frac{\beta c(t)k}{2\varepsilon}+\frac{1}{2}\sqrt{(\frac{\beta c(t)k}{\varepsilon})^{2}+4\frac{\beta c(t)}{d_{iq}+\delta}}\right]\frac{S(t)}{N(t)}$ (S4)

Based on the data of cumulative confirmed cases, cumulative death cases and cumulative cured cases reported by the National Health Commission of China from Jan 10, 2020 to Jan 22, 2020 (Table S2), we reestimated the parameters and initial values of the SEIQR models (S1) and (S2) by using the methods of least square and MCMC, as shown in Table S4.

**Table S4.** Parameters and initial values for models (S1) and (S2) estimated by using the reported data from Jan 10 to Jan 22, 2020 in the mainland of China.

| Parameters | Meanings | Values | 95% CI | References |
| --- | --- | --- | --- | --- |
| $\beta$ | The transmission rate | 0.0197 | (0.0191, 0.0203) | Estimated |
| $\boldsymbol{c}$(*t*) | Contact rate | $c(t)=c_{0}\exp(-\frac{(t-\mu)^{2}}{2\sigma^{2}})$ | | Estimated |
|  | $c_{0}$ | 49.3619 | (48.8976, 49.8262) |  |
|  | $\sigma$ | 86.2796 | (86.2301, 86.3291) |  |
|  | $\mu$ | 15.2306 | (14.7913, 15.6700) |  |
| $k$ | The relative transmission strength of latent individuals to the infectious individuals in the free environment | 0.1917 | (0.1888, 0.1945) | Estimated |
| $d_{iq}$ | The transfer rate from infectious individuals in the free environment to diagnosed individuals | 0.1310 | (0.1280, 0.1340) | Estimated |
| $\delta$ | The death rate due to infection | 0.0022 | (0.0022, 0.0022) | Estimated |
| $\gamma$ | The recovery rate from diagnosed individuals to recovered individuals | 0.0191 | (0.0188, 0.0194) | Estimated |
| $\varepsilon$ | The transfer rate from latent individuals to infectious individuals in the free environment | 1/5.2 | -- | 4 |
| $S(0)$ | The initial value of susceptible individuals in the free environment | 5.9618e+07 | ( 5.9616e+07, 5.9620e+07) | Estimated |
| $E(0)$ | The initial value of latent individuals | 92 | (85, 98) | Estimated |
| $I(0)$ | The initial value of infectious individuals in the free environment | 2 | (1, 4) | Estimated |
| $\boldsymbol{Q}$(*0*) | The initial value of diagnosed individuals | 41 | -- | Reported data |
| $\boldsymbol{R}$(*0*) | The initial value of recovered individuals | 0 | -- | Reported data |
| $\boldsymbol{H}$(*0*) | The initial value of cumulative confirmed cases | 41 | -- | Reported data |
| $\boldsymbol{Z}$(*0*) | The initial value of cumulative deaths | 1 | -- | Reported data |

The comparison between the estimated values by models (S1) and (S2) and the actual reported data was shown in Figure S4. It can be seen that the estimated results of the model fitted well with the actual reported data. In this case, the new SEIQR model (S1) described the natural infectious process of COVID-19, which can be used to predict the natural development trend of the disease in a free environment.

(A)


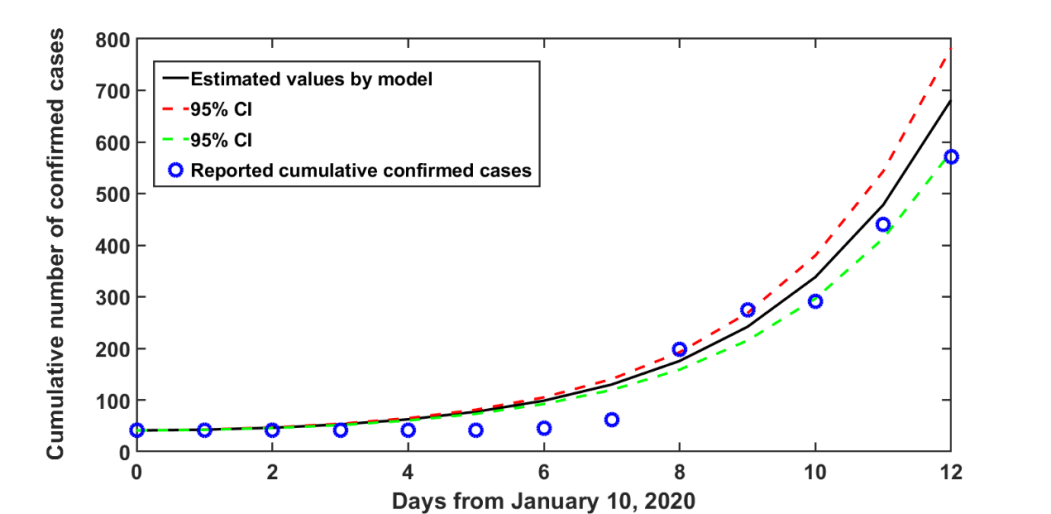


(B)


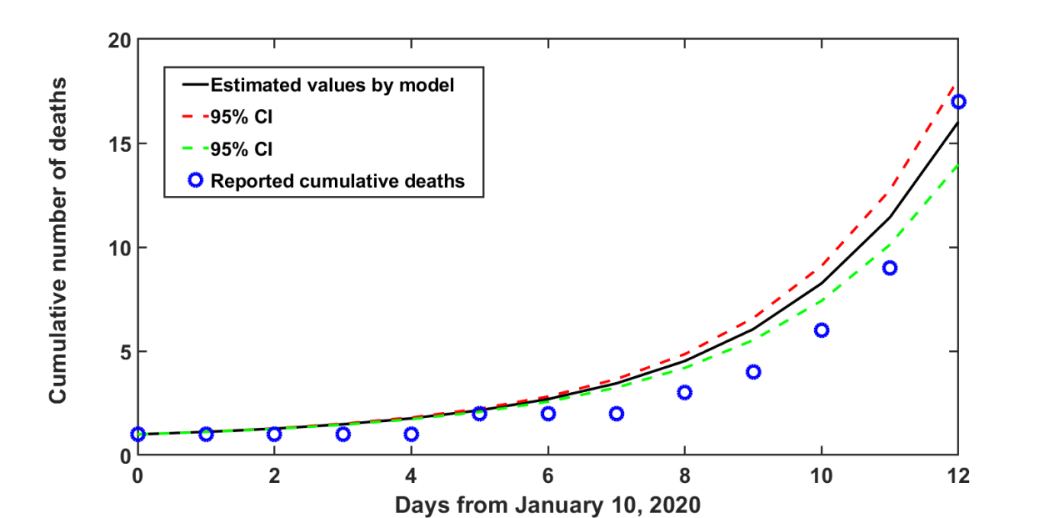


(C)


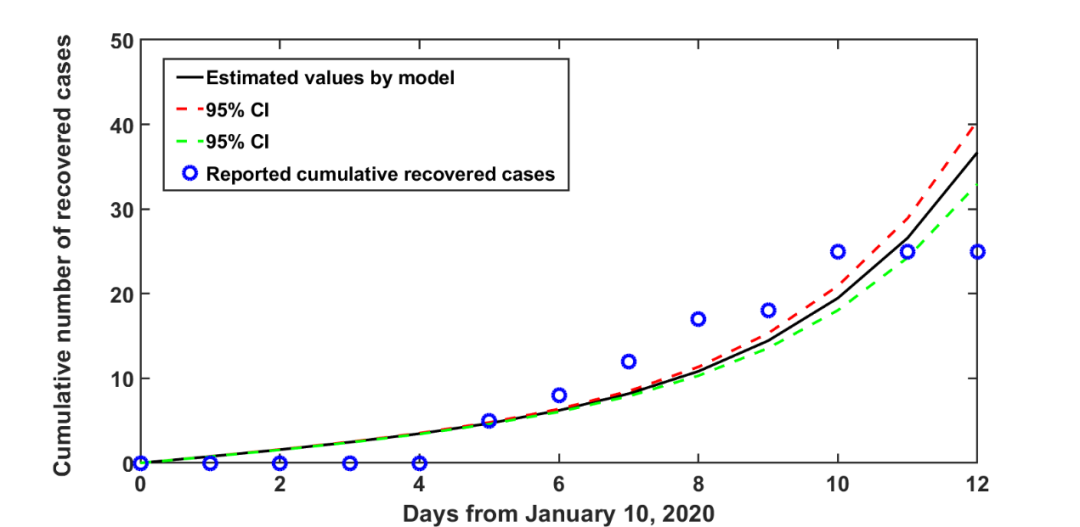


**Figure S4.** Comparison of estimated values by models (S1) and (S2) with real reported data. (A) Cumulative number of confirmed cases over time. (B) Cumulative number of deaths over time. (C) Cumulative number of recovered cases over time. The red dashed line was the upper limit of the 95% CI, and the green dashed line was the lower limit of the 95% CI.

**S.6 Impact of relaxing isolation on the epidemic trend of Hubei Province**

In this section, we reestimated the parameters and initial values of models (1) and (2) based on the reported data (Table S3) from Hubei Province, and analyzed the impact of relaxation quarantine on the epidemic trend of Hubei Province.

**S.6.1 Estimation of parameters and initial values for Hubei Province**

Based on the reported data Table S3, by using the methods of least square and MCMC, we obtained the parameters and initial values for models (1) and (2) in Hubei Province (Table S5).

**Table S5.** Parameters and initial values for models (1) and (2) in Hubei Province.

| Parameters | Meanings | Values | 95% CI | References |
| --- | --- | --- | --- | --- |
| $q$ | The quarantined rate of close contacts | 0.1825 | (0.1819, 0.1830) | Estimated |
| $\beta$ | The transmission rate | 0.0818 | (0.0815, 0.0821) | Estimated |
| $k$ | The relative transmission strength of latent individuals to the infectious individuals in the free environment | 0.9547 | (0.9524, 0.9570) | Estimated |
| $b_{sp}$ | The transfer rate from suspected individuals to susceptible individuals in the free environment | 0.0521 | (0.0517, 0.0525) | Estimated |
| $d_{iq}$ | The transfer rate from infectious individuals in the free environment to diagnosed individuals | 0.1233 | (0.1230, 0.1235) | Estimated |
| $d_{sp}$ | The transfer rate from susceptible individuals in the free environment to suspected individuals | 9.3904e-05 | (0.0782e-03, 0.1096e-03) | Estimated |
| $d_{ep}$ | The transfer rate from latent individuals to suspected individuals | 0.3046 | (0.3040, 0.3052) | Estimated |
| $d_{pq}$ | The transfer rate from suspected individuals to diagnosed individuals | 0.1958 | (0.1947, 0.1968) | Estimated |
| $\boldsymbol{c}$(*t*) | Contact rate | $c(t)=c_{1}+c_{2}\exp(-c_{3}t)$ | | Estimated |
|  | $c_{1}$ | 3.3765e-04 | (0.3105e-03, 0.3647e-03) |  |
|  | $c_{2}$ | 19.5712 | (19.5691, 19.5734) |  |
|  | $c_{3}$ | 0.1293 | (0.1289, 0.1296) |  |
| $\delta$ | The death rate due to infection | 0.0029 | (0.0028, 0.0030) | Estimated |
| $\gamma$ | The recovery rate from diagnosed individuals to recovered individuals | 0.0177 | (0.0176, 0.0178) | Estimated |
| $\lambda$ | The release rate from traced susceptible individuals to susceptible individuals in the free environment | 1/14 | -- | 18 |
| $\varepsilon$ | The transfer rate from latent individuals to infectious individuals in the free environment | 1/5.2 | -- | 4 |
| $S(0)$ | The initial value of susceptible individuals in the free environment | 5.8144e+07 | (5.8129e+07, 5.8159e+07) | Estimated |
| $S_{q}(0)$ | The initial value of traced susceptible individuals | 174 | (170, 179) | Estimated |
| $E(0)$ | The initial value of latent individuals | 152 | (149, 155) | Estimated |
| $E_{q}(0)$ | The initial value of traced latent individuals | 2057 | (2048, 2067) | Estimated |
| $I(0)$ | The initial value of infectious individuals in the free environment | 36 | (33, 40) | Estimated |
| $P(0)$ | The initial value of suspected individuals | 1045 | (1040, 1051) | Estimated |
| $\boldsymbol{Q}$(0) | The initial value of diagnosed individuals | 494 | -- | Reported data |
| $\boldsymbol{R}$(0) | The initial value of recovered individuals | 31 | -- | Reported data |
| $\boldsymbol{H}$(0) | The initial value of cumulative confirmed cases | 549 | -- | Reported data |
| $\boldsymbol{Z}$(0) | The initial value of cumulative deaths | 24 | -- | Reported data |
| *N* | The total population of Hubei Province | 59,170,000 | -- | Reported data |

**S.6.2 Comparison with the daily reported data in Hubei Province**

We compared the model estimation results with 5 different reported data in Table S3. From Figure S5, we can see that the estimated results of the model fitted well with the actual reported data. This comparison served as a validation of the model and parameter values. Thus, models (1) and (2) and the estimated parameters in Table S5 can be used to predict the epidemic trend of COVID-19 in Hubei Province.

(A)


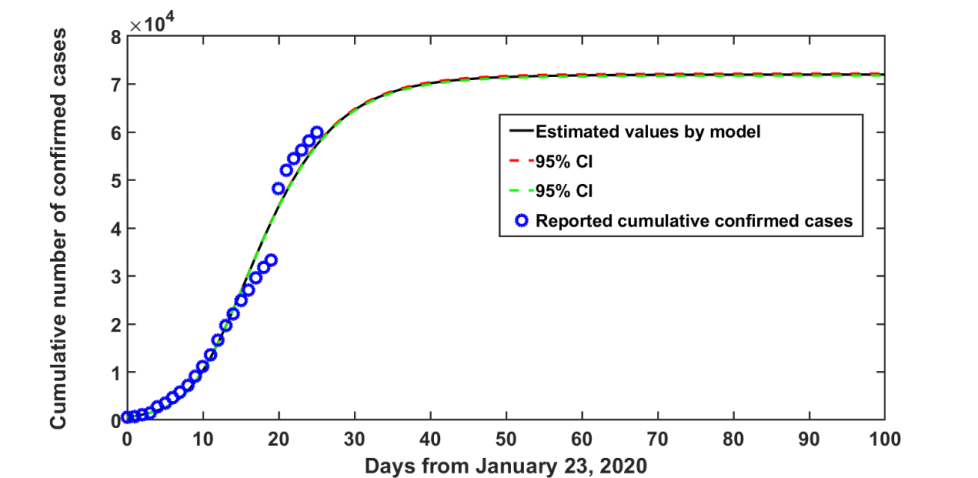


(B)


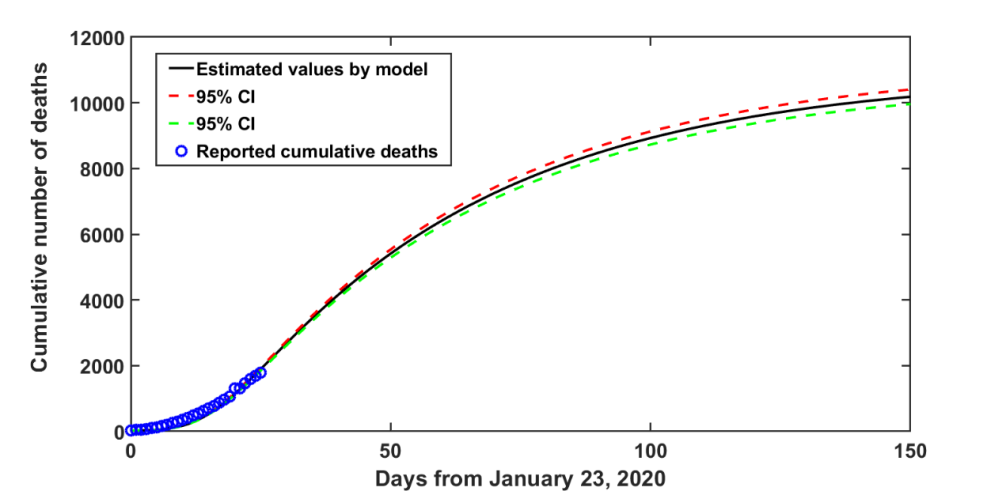


(C)


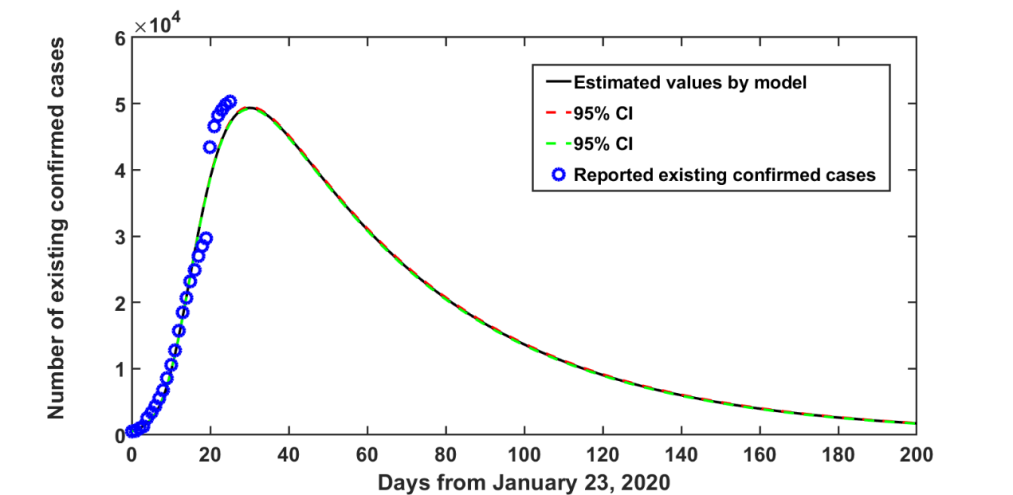


(D)


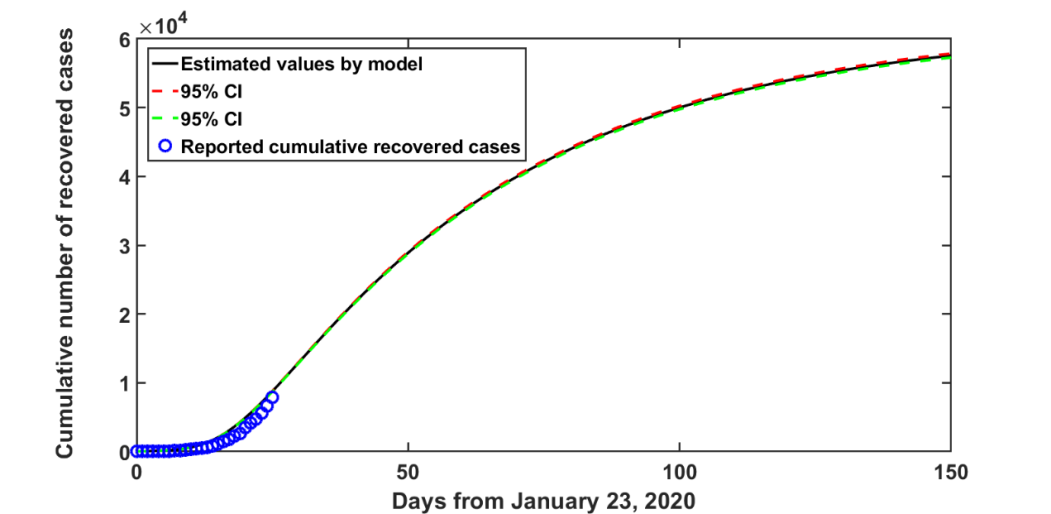


(E)


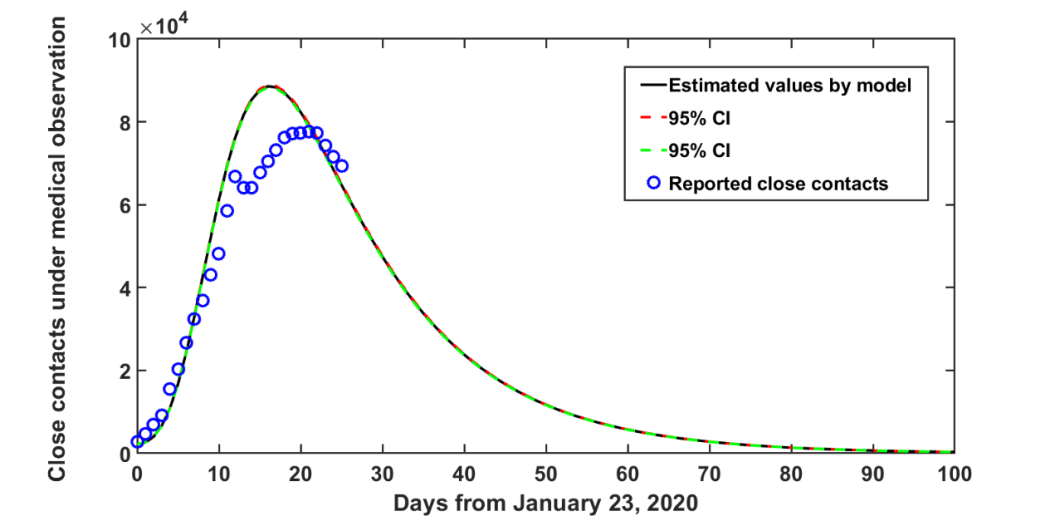


**Figure S5.** Comparison of estimated values by model with real reported data in Hubei Province. (A) Cumulative number of confirmed cases over time. (B) Cumulative number of deaths over time. (C) Number of existing confirmed cases over time. (D) Cumulative number of recovered cases over time. (E) Close contacts under medical observation over time. The red dashed line was the upper limit of the 95% CI, and the green dashed line was the lower limit of the 95% CI.

**S.6.3 Epidemic trend of COVID-19 in Hubei Province**

From Figure S5A, we can see that the finally cumulative number of confirmed cases in Hubei Province will reach to 72,023 (95%CI, 71,815-72,023). The cumulative number of confirmed cases will stabilize from Apr 1, 2020, which indicated that the number of new confirmed cases every day was very small. Since Mar 13, 2020, the number of new confirmed cases will be less than 100 every day, and from Apr 1, 2020, the new confirmed cases will be less than 10 every day. The number of new confirmed cases every day started to decline from Feb 8, 2020. And up to Mar 15, the overall mortality due to disease was approximately 7.87% (95%CI, 7.68%-8.05%) (Figure S5B).

The number of existing confirmed cases in Hubei Province will reach to its peak around Feb 22, 2020 and the number will reach to 49,353 cases (95%CI, 49,201-49,505) (Figure S5C). After the peak, the number of existing confirmed cases will slowly decline, approximately following a Chi-square distribution.

Substitute the parameter values and initial value in Table S5 into equation (3), we obtained the effective reproduction number in Hubei Province (Figure S6A). From Figure S6A, we can see that on Jan 23, 2020, the effective reproduction number of COVID-19 in Hubei Province was about 3.511 (95%CI, 3.489-3.534). On Feb 7, 2020, the effective reproduction number had dropped below 1.0, which suggested that the number of new infections would gradually decline from Feb 7, 2020.

From Figures S6B, C, we can see that the number of free latent people reached to its peak around Feb 3, 2020, with 9,464 cases. The number of free infected people reached to its peak around Feb 9, 2020, with a peak of 9,658 cases, which will drop to 0 by Apr 30, 2020.

(A)


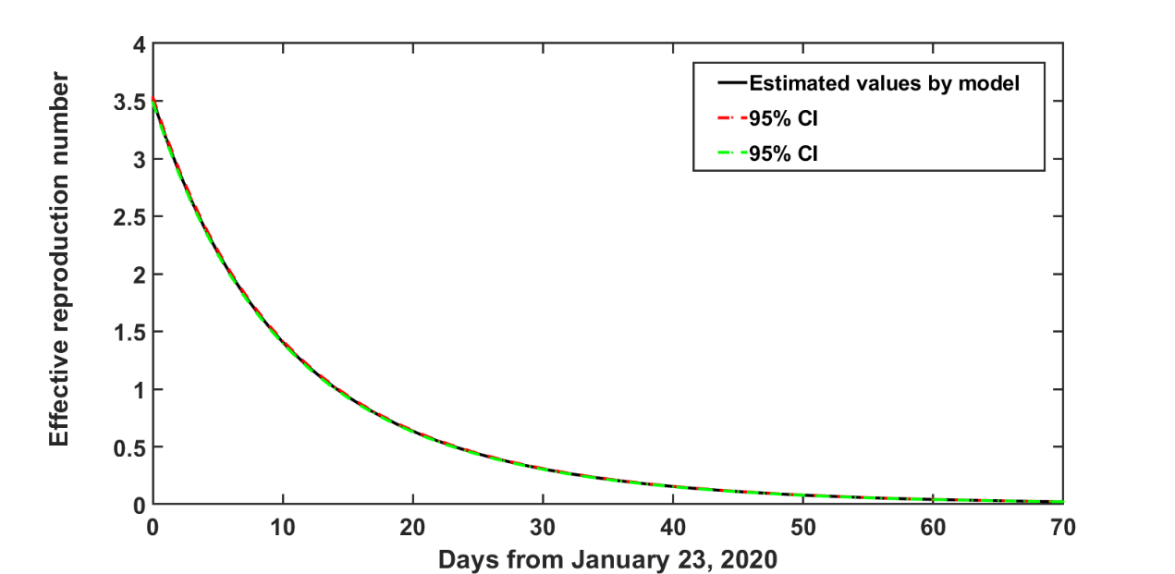


(B)


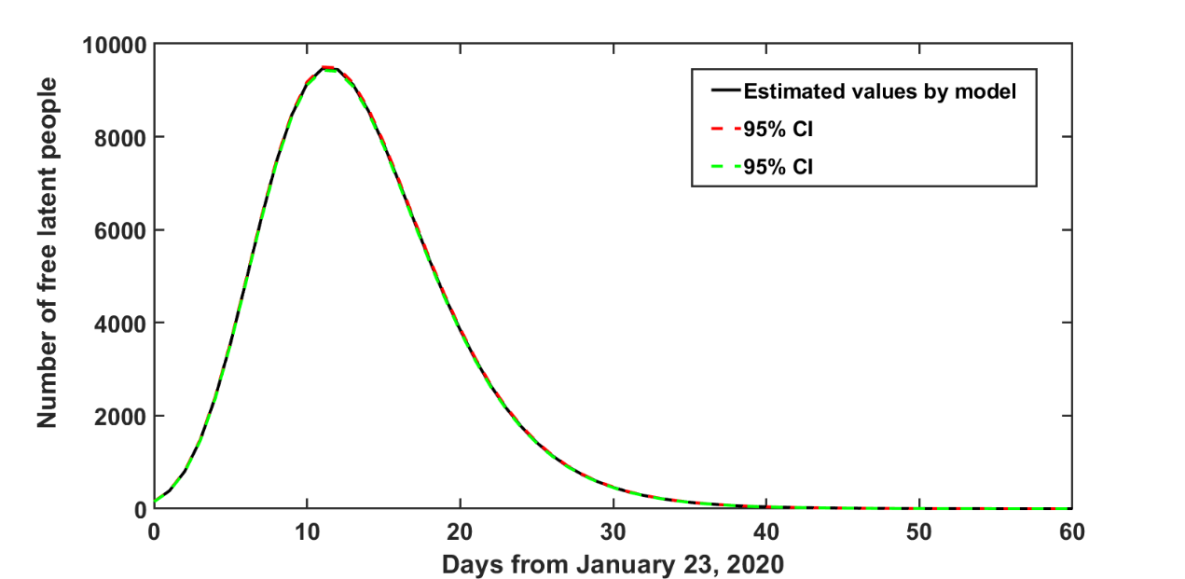


(C)


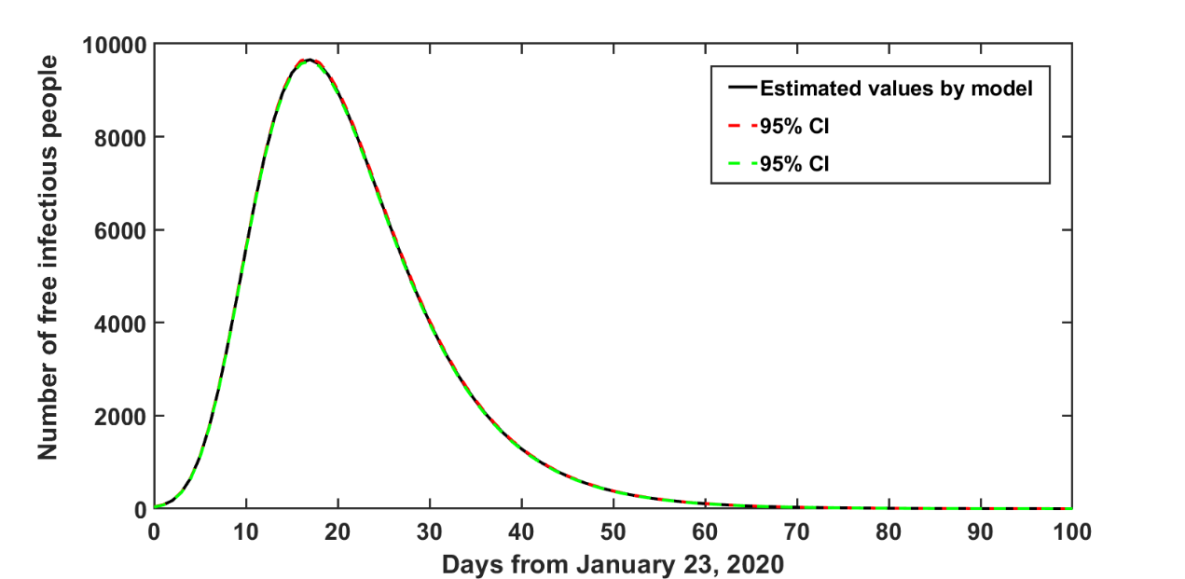


**Figure S6.** Epidemic trend of free infected and latent people. (A) Effective reproduction number in Hubei Province over time. (B) Number of free latent people over time. (C) Number of free infected people over time. The red dashed line was the upper limit of the 95% CI, and the green dashed line was the lower limit of the 95% CI.

**S.6.4 Impact of relaxing isolation on the epidemic trend of COVID-19 in Hubei Province**

If the quarantine was relaxed in Hubei Province from Feb 24, 2020 and the contact rate satisfied *c*>3.0554, then there would be a second peak of infection, when *c*= 3.523, the number of existing confirmed cases will reach to 2,363,000 at the second peak time (Figure S7A).

However, if the quarantine was relaxed from Feb 24, 2020 and the contact rate satisfied *c*≤3.055, then the number of existing confirmed cases would continue to decline, but the downward trend would be slower (when *c*=3.034, shown in Figure S7B). From Figures S7B, C, D and Table S6, we can see that when *c*= 3.034, delaying the time of relaxation isolation will greatly reduce the disease-related mortality and cumulative confirmed cases. Compared with the current situation, if isolation was relaxed on Apr 1, then on Apr 30 the number of existing confirmed cases will only increase by 3.36%; the cumulative number of confirmed cases and deaths will only crease by 0.02% and 0.17%, respectively. Based on the above analysis, we proposed to gradually relax the quarantine from Apr 1 in Hubei Province.

(A)


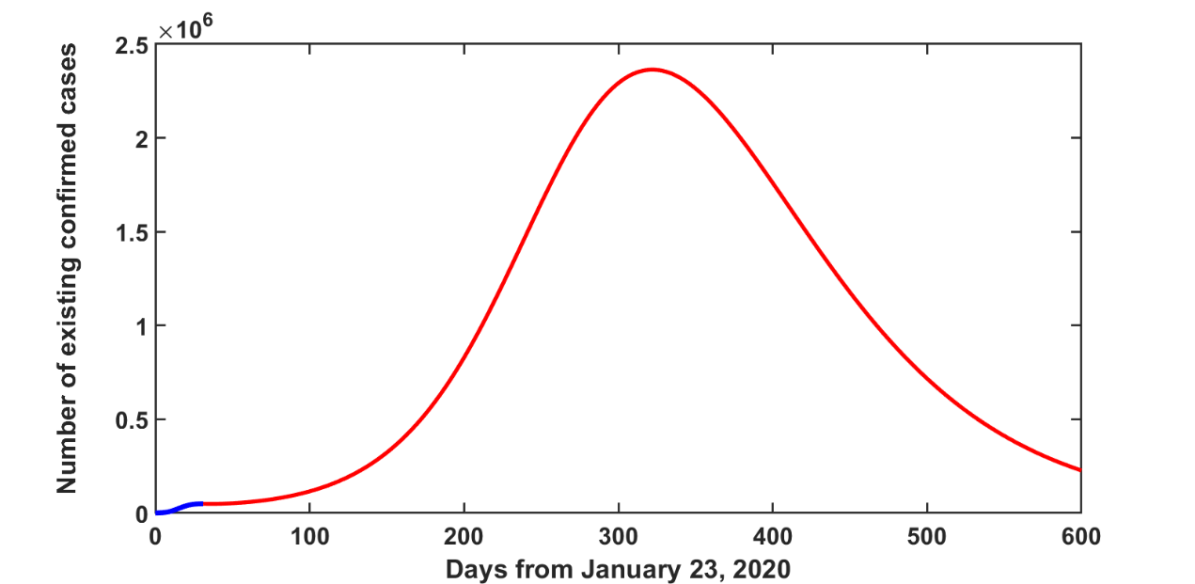


(B)


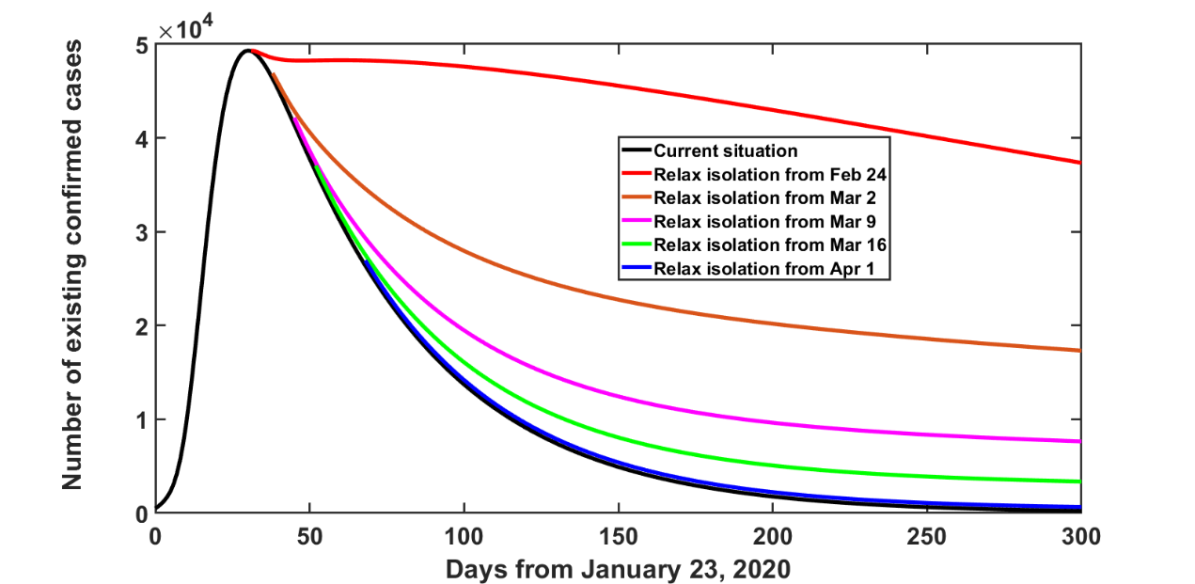


(C)


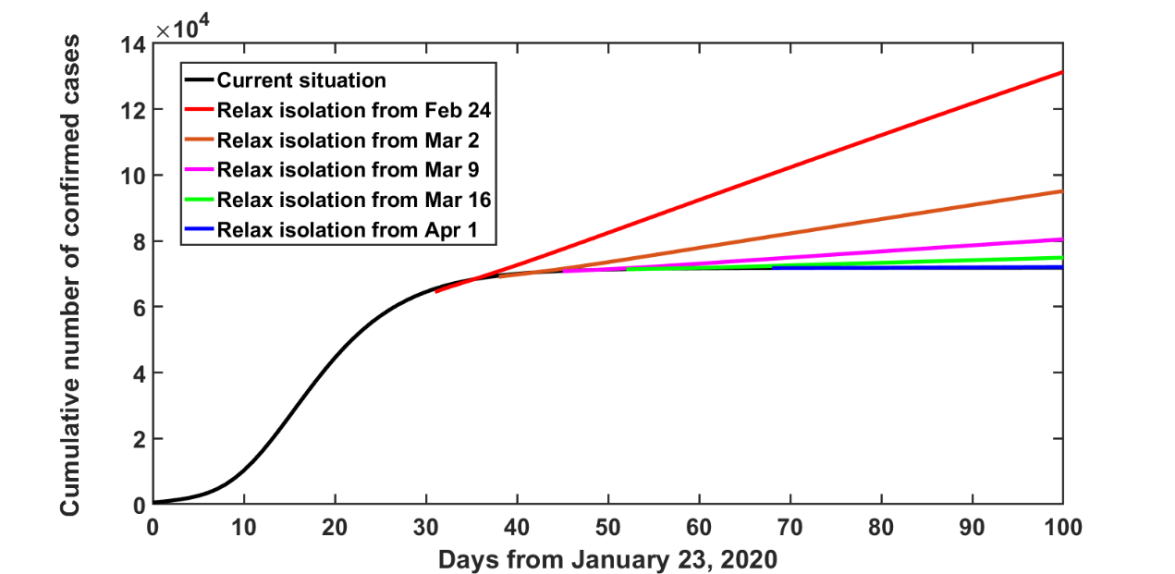


(D)


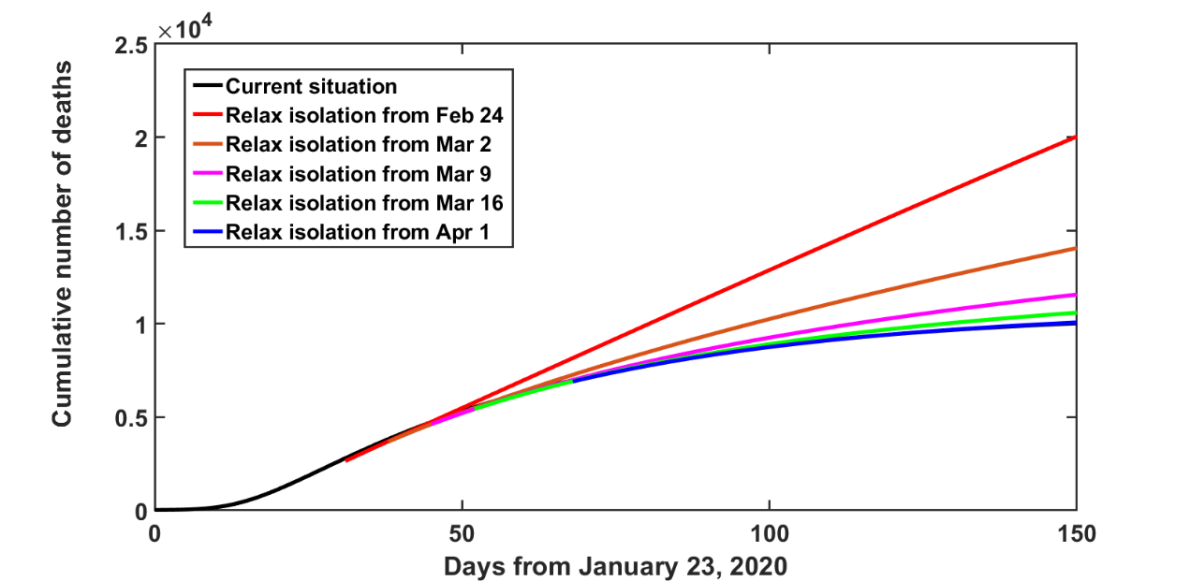


**Figure S7.** Impact of the relaxing quarantine at different times on the epidemic trend of COVID-19 in Hubei Province. (A) On the number of existing confirmed cases when *c*= 3.523. (B) On the number of existing confirmed cases when *c*=3.034.(C) On the cumulative number of confirmed cases when *c*=3.034.(D) On the cumulative number of deaths when *c*=3.034.

**Table S6.** Impact of relaxing isolation at different times on the epidemic trend of COVID-19 in Hubei Province on Apr 30, 2020 compared to the current situation (*c*=3.034).

| Relaxing isolation date | Percentage increase in cumulative confirmed cases | Percentage increase in cumulative deaths | Percentage increase in existing confirmed cases |
| --- | --- | --- | --- |
| 2020/2/24 | 79.67% | 45.47% | 233.75% |
| 2020/3/2 | 30.93% | 16.58% | 97.90% |
| 2020/3/9 | 11.22% | 5.63% | 39.57% |
| 2020/3/16 | 3.77% | 1.84% | 16.18% |
| 2020/4/1 | 0.02% | 0.17% | 3.36% |

**S.7 The Matlab code for estimation of parameters and initial values by using the least square method**

function NLSQUANGUO20200229

clear all

clc

lb=[0 0 0 0 0 1/7 0 0 0 0 0 0 0 0 0 0 0 0 0]; % Constraint condition, the lower limit of parameters

ub=[1 1 20 1 1 1 1 1 1 1 1 25000 20000 25000 1400000000 20000 20 1 10000]; % Constraint condition, the upper limit of parameters

par1guess=[0.5 0.05 5 0.1 0.03 1/7.6 0.06 1/5.2 0.695/7.6 0.021/8 0.1 2.0000e+04 7.1827e+03 2.0000e+04 1.3500e+09 3.8845e+03 9 0.13 1072];% The initial value of parameters

options=optimset('Algorithm','trust-region-reflective','Display','final','MaxIter',100000,'MaxFunEvals',100000); % Optimization options

[par1,resnorm,residual,exitflag,output,lambda,jacobian]=lsqnonlin(@LSminzu,par1guess,lb,ub,options);

Estimated_Paras=par1 % Estimated parameters

RMSE=sqrt(resnorm/182)

sigma_par1=std(par1);

par1_ci=[par1-1.96*sigma_par1; par1+1.96*sigma_par1]'

q=par1(1)

beta=par1(2)

c1=par1(3)

k=par1(4)

bsp=par1(5)

diq=par1(6)

dsp=par1(7)

dep=par1(8)

dpq=par1(9)

dita=par1(10)

recov=par1(11)

E0=par1(12)

I0=par1(13)

Eq0=par1(14)

S0=par1(15)

Sq0=par1(16)

c2=par1(17)

c3=par1(18)

P0=par1(19)

% Comparison of estimated values by models (1) and (2) with reported data

Ic=[S0 Sq0 E0 Eq0 I0 P0 771 34 830 1072 25]; % Initial conditions

[T,W]=ode45(@xyvmodel,[0:1:200],Ic,[],par1);

[T2,W2]=ode45(@xyvmodel,[0:1:25],Ic,[],par1);

[T3,W3]=ode45(@xyvmodel,[0:1:200],Ic,[],par1);

Z1=W3(:,9)';Z2=W3(:,6)';Z3=W3(:,2)'+W3(:,4)';

J1=Z1(1,1:1:200);

J2=Z2(1,1:1:200);

J3=Z3(1,1:1:200);

save('H.mat','J1')

save('P.mat','J2')

save('SqEq.mat','J3')

figure(1) % S(t)

plot(T,W(:,1),'k-','LineWidth',4);

xlabel('Days from January 23, 2020');

ylabel('Number of S');

figure(2) % Sq(t)

plot(T,W(:,2),'k-','LineWidth',4);

xlabel('Days from January 23, 2020');

ylabel('Number of Sq');

figure(3) % E(t)

plot(T,W(:,3),'k-','LineWidth',4);

xlabel('Days from January 23, 2020');

ylabel('Number of E');

figure(4) % Eq(t)

plot(T,W(:,4),'k-','LineWidth',4);

xlabel('Days from January 23, 2020');

ylabel('Number of Eq');

figure(5) % I(t)

plot(T,W(:,5),'k-','LineWidth',4);

xlabel('Days from January 23, 2020');

ylabel('Number of I');

figure(6) % P(t)

plot(T2,W2(:,6),'k-','LineWidth',4);

hold on

plot([0 1 2 3 4 5 6 7 8 9 10 11 12 13 14 15 16 17 18 19 20 21 22 23 24 25],[1072 1965 2684 5794 6973 9239 12167 15238 17988 19544 21558 23214 23260 24702 26359 27657 28942 23589 21675 16067 13435 10109 8969 8228 7264 6242],'bo','LineWidth',2,'MarkerSize',9);

xlabel('Days from January 23, 2020');

ylabel('Number of P');

figure(7) % P(t)

plot(T,W(:,6),'k-','LineWidth',4);

hold on

plot([0 1 2 3 4 5 6 7 8 9 10 11 12 13 14 15 16 17 18 19 20 21 22 23 24 25],[1072 1965 2684 5794 6973 9239 12167 15238 17988 19544 21558 23214 23260 24702 26359 27657 28942 23589 21675 16067 13435 10109 8969 8228 7264 6242],'bo','LineWidth',2,'MarkerSize',9);

xlabel('Days from January 23, 2020');

ylabel('Number of P');

figure(8) % Q(t)

plot(T2,W2(:,7),'k-','LineWidth',4);

hold on

plot([0 1 2 3 4 5 6 7 8 9 10 11 12 13 14 15 16 17 18 19 20 21 22 23 24 25],[771 1208 1870 2613 4349 5739 7417 9308 11289 13748 16369 19381 22942 26302 28985 31774 33738 35982 37626 38800 52526 55748 56873 57416 57934 58016],'bo','LineWidth',2,'MarkerSize',9);

xlabel('Days from January 23, 2020');

ylabel('Number of Q');

figure(9) % Q(t)

plot(T,W(:,7),'k-','LineWidth',4);

hold on

plot([0 1 2 3 4 5 6 7 8 9 10 11 12 13 14 15 16 17 18 19 20 21 22 23 24 25],[771 1208 1870 2613 4349 5739 7417 9308 11289 13748 16369 19381 22942 26302 28985 31774 33738 35982 37626 38800 52526 55748 56873 57416 57934 58016],'bo','LineWidth',2,'MarkerSize',9);

xlabel('Days from January 23, 2020');

ylabel('Number of Q');

figure(10) % R(t)

plot(T,W(:,8),'k-','LineWidth',4);

hold on

plot([0 1 2 3 4 5 6 7 8 9 10 11 12 13 14 15 16 17 18 19 20 21 22 23 24 25],[34 38 49 51 60 103 124 171 243 328 475 632 892 1153 1540 2050 2649 3281 3996 4740 5911 6723 8096 9419 10844 12552],'bo','LineWidth',2,'MarkerSize',9);

xlabel('Days from January 23, 2020');

ylabel('Number of R');

figure(11) % R(t)

plot(T2,W2(:,8),'k-','LineWidth',4);

hold on

plot([0 1 2 3 4 5 6 7 8 9 10 11 12 13 14 15 16 17 18 19 20 21 22 23 24 25],[34 38 49 51 60 103 124 171 243 328 475 632 892 1153 1540 2050 2649 3281 3996 4740 5911 6723 8096 9419 10844 12552],'bo','LineWidth',2,'MarkerSize',9);

xlabel('Days from January 23, 2020');

ylabel('Number of R');

figure(12) % H(t)

plot(T,W(:,9),'k-','LineWidth',4);

hold on

plot([0 1 2 3 4 5 6 7 8 9 10 11 12 13 14 15 16 17 18 19 20 21 22 23 24 25],[830 1287 1975 2744 4515 5974 7711 9692 11791 14380 17205 20438 24324 28018 31161 34546 37198 40171 42638 44653 59804 63851 66492 68500 70548 72436],'bo','LineWidth',2,'MarkerSize',9);

xlabel('Days from January 23, 2020');

ylabel('Number of H');

figure(13) % Y(t)

plot(T2,W2(:,10),'k-','LineWidth',4);

hold on

plot([0 1 2 3 4 5 6 7 8 9 10 11 12 13 14 15 16 17 18 19 20 21 22 23 24 25],[1072 2190 3499 7305 9382 12630 16778 21590 26609 31171 36344 41416 45387 50715 55548 59762 63678 67686 71222 74564 77371 79821 82098 84016 85579 87011],'bo','LineWidth',2,'MarkerSize',9);

xlabel('Days from January 23, 2020');

ylabel('Number of Y');

figure(14) % Y(t)

plot(T,W(:,10),'k-','LineWidth',4);

hold on

plot([0 1 2 3 4 5 6 7 8 9 10 11 12 13 14 15 16 17 18 19 20 21 22 23 24 25],[1072 2190 3499 7305 9382 12630 16778 21590 26609 31171 36344 41416 45387 50715 55548 59762 63678 67686 71222 74564 77371 79821 82098 84016 85579 87011],'bo','LineWidth',2,'MarkerSize',9);

xlabel('Days from January 23, 2020');

ylabel('Number of Y');

figure(15) % Z(t)

plot(T2,W2(:,11),'k-','LineWidth',4);

hold on

plot([0 1 2 3 4 5 6 7 8 9 10 11 12 13 14 15 16 17 18 19 20 21 22 23 24 25],[25 41 56 80 106 132 170 213 259 304 361 425 490 563 636 722 811 908 1016 1113 1367 1380 1523 1665 1770 1868],'bo','LineWidth',2,'MarkerSize',9);

xlabel('Days from January 23, 2020');

ylabel('Number of Z');

figure(16) % Z(t)

plot(T,W(:,11),'k-','LineWidth',4);

hold on

plot([0 1 2 3 4 5 6 7 8 9 10 11 12 13 14 15 16 17 18 19 20 21 22 23 24 25],[25 41 56 80 106 132 170 213 259 304 361 425 490 563 636 722 811 908 1016 1113 1367 1380 1523 1665 1770 1868],'bo','LineWidth',2,'MarkerSize',9);

xlabel('Days from January 23, 2020');

ylabel('Number of Z');

figure(17) % Sq(t)+Eq(t)

plot(T2,W2(:,2)+W2(:,4),'k-','LineWidth',4);

hold on

plot([0 1 2 3 4 5 6 7 8 9 10 11 12 13 14 15 16 17 18 19 20 21 22 23 24 25],[8420 13967 21556 30453 44132 59990 81947 102427 118478 137594 152700 171329 185555 186354 186045 189660 188183 187518 187728 185037 181386 177984 169039 158764 150539 141552],'bo','LineWidth',2,'MarkerSize',9);

xlabel('Days from January 23, 2020');

ylabel('Number of Sq+Eq');

figure(18) % Sq(t)+Eq(t)

plot(T,W(:,2)+W(:,4),'k-','LineWidth',4);

hold on

plot([0 1 2 3 4 5 6 7 8 9 10 11 12 13 14 15 16 17 18 19 20 21 22 23 24 25],[8420 13967 21556 30453 44132 59990 81947 102427 118478 137594 152700 171329 185555 186354 186045 189660 188183 187518 187728 185037 181386 177984 169039 158764 150539 141552],'bo','LineWidth',2,'MarkerSize',9);

xlabel('Days from January 23, 2020');

ylabel('Number of Sq+Eq');

% Define the objective function of NLS

function diff=LSminzu(par1,Y)

lambda=1/14;

epsilon=1/5.2;

q=par1(1);

beta=par1(2);

c1=par1(3);

k=par1(4);

bsp=par1(5);

diq=par1(6);

dsp=par1(7);

dep=par1(8);

dpq=par1(9);

dita=par1(10);

recov=par1(11);

E0=par1(12);

I0=par1(13);

Eq0=par1(14);

S0=par1(15);

Sq0=par1(16);

c2=par1(17);

c3=par1(18);

P0=par1(19); % The initial values of parameters

Ic=[S0 Sq0 E0 Eq0 I0 P0 771 34 830 1072 25]; % Initial conditions

[T,W]=ode45(@xyvmodel,[0:1:200],Ic,[],par1);

A7=W(:,6)';A8=W(:,2)'+W(:,4)';A9=W(:,7)';A10=W(:,9)';A11=W(:,10)';A12=W(:,8)';A13=W(:,11)';

B2=A7(1,1:1:26);B3=0.1.*A8(1,1:1:26);B4=A9(1,1:1:26);B5=A10(1,1:1:26);B6=A11(1,1:1:26);B7=A12(1,1:1:26);B8=A13(1,1:1:26);

B=[B2,B3,B4,B5,B6,B7,B8];

Y=[1072 1965 2684 5794 6973 9239 12167 15238 17988 19544 21558 23214 23260 24702 26359 27657 28942 23589 21675 16067 13435 10109 8969 8228 7264 6242 0.1*8420 0.1*13967 0.1*21556 0.1*30453 0.1*44132 0.1*59990 0.1*81947 0.1*102427 0.1*118478 0.1*137594 0.1*152700 0.1*171329 0.1*185555 0.1*186354 0.1*186045 0.1*189660 0.1*188183 0.1*187518 0.1*187728 0.1*185037 0.1*181386 0.1*177984 0.1*169039 0.1*158764 0.1*150539 0.1*141552 771 1208 1870 2613 4349 5739 7417 9308 11289 13748 16369 19381 22942 26302 28985 31774 33738 35982 37626 38800 52526 55748 56873 57416 57934 58016 830 1287 1975 2744 4515 5974 7711 9692 11791 14380 17205 20438 24324 28018 31161 34546 37198 40171 42638 44653 59804 63851 66492 68500 70548 72436 1072 2190 3499 7305 9382 12630 16778 21590 26609 31171 36344 41416 45387 50715 55548 59762 63678 67686 71222 74564 77371 79821 82098 84016 85579 87011 34 38 49 51 60 103 124 171 243 328 475 632 892 1153 1540 2050 2649 3281 3996 4740 5911 6723 8096 9419 10844 12552 25 41 56 80 106 132 170 213 259 304 361 425 490 563 636 722 811 908 1016 1113 1367 1380 1523 1665 1770 1868]; %Reported data

diff=B-Y;

% Definition of the models (1) and (2)

dw=zeros(11,1);

function dw=xyvmodel(t,w,par1)

lambda=1/14;

epsilon=1/5.2;

q=par1(1);

beta=par1(2);

c1=par1(3);

k=par1(4);

bsp=par1(5);

diq=par1(6);

dsp=par1(7);

dep=par1(8);

dpq=par1(9);

dita=par1(10);

recov=par1(11);

E0=par1(12);

I0=par1(13);

Eq0=par1(14);

S0=par1(15);

Sq0=par1(16);

c2=par1(17);

c3=par1(18);

P0=par1(19);

dw=[-((1- beta)*q+beta)*(c1+c2*exp(-c3*t))*w(1)*(k*w(3)+w(5))/1400000000-dsp*w(7)+bsp*w(6)+lambda*w(2);

(1-beta)*q*(c1+c2*exp(-c3*t))*w(1)*(k*w(3)+w(5))/1400000000-lambda*w(2);

(1-q)*beta*(c1+c2*exp(-c3*t))*w(1)*(k*w(3)+w(5))/1400000000-epsilon*w(3)-dep*w(3);

q*beta*(c1+c2*exp(-c3*t))*w(1)*(k*w(3)+w(5))/1400000000-dep*w(4);

epsilon*w(3)-diq*w(5)-dita*w(5);

dsp*w(7)+dep*w(4)+dep*w(3)-bsp*w(6)-dpq*w(6);

dpq*w(6)-recov*w(7)-dita*w(7)+diq*w(5);

recov*w(7);

diq*w(5)+dpq*w(6);

dsp*w(7)+dep*w(4)+dep*w(3);

dita*w(5)+dita*w(7)];

**S.8 The Matlab code for estimation of parameters and initial values by using MCMC approach.**

**% Step 1**

function MCMCQUANGUO23H02291

clear all

clc

Y=[1072 1965 2684 5794 6973 9239 12167 15238 17988 19544 21558 23214 23260 24702 26359 27657 28942 23589 21675 16067 13435 10109 8969 8228 7264 6242 0.1*8420 0.1*13967 0.1*21556 0.1*30453 0.1*44132 0.1*59990 0.1*81947 0.1*102427 0.1*118478 0.1*137594 0.1*152700 0.1*171329 0.1*185555 0.1*186354 0.1*186045 0.1*189660 0.1*188183 0.1*187518 0.1*187728 0.1*185037 0.1*181386 0.1*177984 0.1*169039 0.1*158764 0.1*150539 0.1*141552 771 1208 1870 2613 4349 5739 7417 9308 11289 13748 16369 19381 22942 26302 28985 31774 33738 35982 37626 38800 52526 55748 56873 57416 57934 58016 830 1287 1975 2744 4515 5974 7711 9692 11791 14380 17205 20438 24324 28018 31161 34546 37198 40171 42638 44653 59804 63851 66492 68500 70548 72436 1072 2190 3499 7305 9382 12630 16778 21590 26609 31171 36344 41416 45387 50715 55548 59762 63678 67686 71222 74564 77371 79821 82098 84016 85579 87011 34 38 49 51 60 103 124 171 243 328 475 632 892 1153 1540 2050 2649 3281 3996 4740 5911 6723 8096 9419 10844 12552 25 41 56 80 106 132 170 213 259 304 361 425 490 563 636 722 811 908 1016 1113 1367 1380 1523 1665 1770 1868]; % Reported data

q = 0.2644;

beta = 0.0776;

c1 = 5.0300e-05;

k = 0.5833;

bsp = 0.0872;

diq = 0.1510;

dsp = 3.2554e-05;

dep = 0.3104;

dpq = 0.1132;

dita = 0.0021;

recov = 0.0161;

E0 = 1.1790e+03;

I0 = 786.2891;

Eq0 = 2.6553e+03;

S0 = 1.3872e+09;

Sq0 = 789.9653;

c2 = 19.6325;

c3 = 0.1081;

P0 = 1.8787e+03; % The initial values of parameters

Olik=Loglik(q,beta,c1,k,bsp,diq,dsp,dep,dpq,dita,recov,E0,I0,Eq0,S0,Sq0,c2,c3,P0,Y);

% Calculate the Logarithm likelihood function

w=[0.001,0.001,0.1,0.001,0.001,0.105,0.00001,0.0001,0.0001,0.001,0.001,5,5,5,1000000,5,0.2,0.01,5]; % The maximum range of random walks

N=8000;m=6000; % MCMC cycle index and burn-in periods

MHPar=zeros(N+1,19);

MHPar(1,:)=[q,beta,c1,k,bsp,diq,dsp,dep,dpq,dita,recov,E0,I0,Eq0,S0,Sq0,c2,c3,P0]; % MCMC algorithm and Random walks to update parameters

for i=2:N+1

NPar=[q,beta,c1,k,bsp,diq,dsp,dep,dpq,dita,recov,E0,I0,Eq0,S0,Sq0,c2,c3,P0]+w.*(2*rand(1,19)-1); % Generate candidate parameters randomly

Nq=NPar(1);Nbeta=NPar(2);Nc1=NPar(3);Nk=NPar(4);Nbsp=NPar(5);Ndiq=NPar(6);

Ndsp=NPar(7);Ndep=NPar(8);Ndpq=NPar(9);Ndita=NPar(10);Nrecov=NPar(11);

NE0=NPar(12);NI0=NPar(13);NEq0=NPar(14);NS0=NPar(15);NSq0=NPar(16);Nc2=NPar(17);Nc3=NPar(18);NP0=NPar(19);

if(Nq>0&&Nbeta>0&&Nc1>0&&Nk>0&&Nbsp>0&&Ndiq>0&&Ndsp>0&&Ndep>0&&Ndpq>0&&Ndita>0&&Nrecov>0&&NE0>0&&NI0>0&&NEq0>0&&NS0>0&&NSq0>0&&Nc2>0&&Nc3>0&&NP0>0) % Accept or reject

Nlik=Loglik(Nq,Nbeta,Nc1,Nk,Nbsp,Ndiq,Ndsp,Ndep,Ndpq,Ndita,Nrecov,NE0,NI0,NEq0,NS0,NSq0,Nc2,Nc3,NP0,Y);

alpha=min(1,exp(Nlik-Olik));

if rand<alpha

q=Nq;beta=Nbeta;c1=Nc1;k=Nk;bsp=Nbsp;diq=Ndiq;dsp=Ndsp;dep=Ndep;dpq=Ndpq;dita=Ndita;recov=Nrecov;

E0=NE0;I0=NI0;Eq0=NEq0;S0=NS0;Sq0=NSq0;c2=Nc2;c3=Nc3;P0=NP0;

Olik=Nlik;

end

end

MHPar(i,:)=[q,beta,c1,k,bsp,diq,dsp,dep,dpq,dita,recov,E0,I0,Eq0,S0,Sq0,c2,c3,P0];

end

Yu=MHPar(m+1:N,:);

save('Yu.mat','Yu')

% Calculate the estimated values of parameters

q=mean(MHPar(m:end,1))

sigma_q=std(MHPar(m:end,1));

q_ci=[q-1.96*sigma_q, q+1.96*sigma_q]

beta=mean(MHPar(m:end,2))

sigma_beta=std(MHPar(m:end,2));

beta_ci=[beta-1.96*sigma_beta, beta+1.96*sigma_beta]

c1=mean(MHPar(m:end,3))

sigma_c1=std(MHPar(m:end,3));

c1_ci=[c1-1.96*sigma_c1, c1+1.96*sigma_c1]

k=mean(MHPar(m:end,4))

sigma_k=std(MHPar(m:end,4));

k_ci=[k-1.96*sigma_k, k+1.96*sigma_k]

bsp=mean(MHPar(m:end,5))

sigma_bsp=std(MHPar(m:end,5));

bsp_ci=[bsp-1.96*sigma_bsp, bsp+1.96*sigma_bsp]

diq=mean(MHPar(m:end,6))

sigma_diq=std(MHPar(m:end,6));

diq_ci=[diq-1.96*sigma_diq, diq+1.96*sigma_diq]

dsp=mean(MHPar(m:end,7))

sigma_dsp=std(MHPar(m:end,7));

dsp_ci=[dsp-1.96*sigma_dsp, dsp+1.96*sigma_dsp]

dep=mean(MHPar(m:end,8))

sigma_dep=std(MHPar(m:end,8));

dep_ci=[dep-1.96*sigma_dep, dep+1.96*sigma_dep]

dpq=mean(MHPar(m:end,9))

sigma_dpq=std(MHPar(m:end,9));

dpq_ci=[dpq-1.96*sigma_dpq, dpq+1.96*sigma_dpq]

dita=mean(MHPar(m:end,10))

sigma_dita=std(MHPar(m:end,10));

dita_ci=[dita-1.96*sigma_dita, dita+1.96*sigma_dita]

recov=mean(MHPar(m:end,11))

sigma_recov=std(MHPar(m:end,11));

recov_ci=[recov-1.96*sigma_recov,recov+1.96*sigma_recov]

E0=mean(MHPar(m:end,12))

sigma_E0=std(MHPar(m:end,12));

E0_ci=[E0-1.96*sigma_E0, E0+1.96*sigma_E0]

I0=mean(MHPar(m:end,13))

sigma_I0=std(MHPar(m:end,13));

I0_ci=[I0-1.96*sigma_I0, I0+1.96*sigma_I0]

Eq0=mean(MHPar(m:end,14))

sigma_Eq0=std(MHPar(m:end,14));

Eq0_ci=[Eq0-1.96*sigma_Eq0, Eq0+1.96*sigma_Eq0]

S0=mean(MHPar(m:end,15))

sigma_S0=std(MHPar(m:end,15));

S0_ci=[S0-1.96*sigma_S0, S0+1.96*sigma_S0]

Sq0=mean(MHPar(m:end,16))

sigma_Sq0=std(MHPar(m:end,16));

Sq0_ci=[Sq0-1.96*sigma_Sq0, Sq0+1.96*sigma_Sq0]

c2=mean(MHPar(m:end,17))

sigma_c2=std(MHPar(m:end,17));

c2_ci=[c2-1.96*sigma_c2, c2+1.96*sigma_c2]

c3=mean(MHPar(m:end,18))

sigma_c3=std(MHPar(m:end,18));

c1_c3=[c3-1.96*sigma_c3, c3+1.96*sigma_c3]

P0=mean(MHPar(m:end,19))

sigma_P0=std(MHPar(m:end,19));

P0_ci=[P0-1.96*sigma_P0, P0+1.96*sigma_P0]

M=[mean(MHPar(m:end,1));mean(MHPar(m:end,2));mean(MHPar(m:end,3));

mean(MHPar(m:end,4));mean(MHPar(m:end,5));mean(MHPar(m:end,6));

mean(MHPar(m:end,7));mean(MHPar(m:end,8));mean(MHPar(m:end,9));

mean(MHPar(m:end,10));mean(MHPar(m:end,11));mean(MHPar(m:end,12));

mean(MHPar(m:end,13));mean(MHPar(m:end,14));mean(MHPar(m:end,15));

mean(MHPar(m:end,16));mean(MHPar(m:end,17));mean(MHPar(m:end,18));mean(MHPar(m:end,19))]';

FangchaM=[std(MHPar(m:end,1));std(MHPar(m:end,2));std(MHPar(m:end,3));

std(MHPar(m:end,4));std(MHPar(m:end,5));std(MHPar(m:end,6));

std(MHPar(m:end,7));std(MHPar(m:end,8));std(MHPar(m:end,9));

std(MHPar(m:end,10));std(MHPar(m:end,11));std(MHPar(m:end,12));

std(MHPar(m:end,13));std(MHPar(m:end,14));std(MHPar(m:end,15));

std(MHPar(m:end,16));std(MHPar(m:end,17));std(MHPar(m:end,18));std(MHPar(m:end,19))]';

% Calculate the Logarithm likelihood function

function [NLik]=Loglik(q,beta,c1,k,bsp,diq,dsp,dep,dpq,dita,recov,E0,I0,Eq0,S0,Sq0,c2,c3,P0,Y)

n=realpop(q,beta,c1,k,bsp,diq,dsp,dep,dpq,dita,recov,E0,I0,Eq0,S0,Sq0,c2,c3,P0);

NLik=sum(log(n).*Y-n-gammaln(Y+1));

end

% Definition of the models (1) and (2)

function [n]=realpop(q,beta,c1,k,bsp,diq,dsp,dep,dpq,dita,recov,E0,I0,Eq0,S0,Sq0,c2,c3,P0)

lambda=1/14;

epsilon=1/5.2;

F=@(t,w)[-((1- beta)*q+ beta)*(c1+c2*exp(-c3*t))*w(1)*(k*w(3)+w(5))/1400000000-dsp*w(7)+bsp*w(6)+lambda*w(2);

(1-beta)*q*(c1+c2*exp(-c3*t))*w(1)*(k*w(3)+w(5))/1400000000-lambda*w(2);

(1-q)*beta*(c1+c2*exp(-c3*t))*w(1)*(k*w(3)+w(5))/1400000000-epsilon*w(3)-dep*w(3);

q*beta*(c1+c2*exp(-c3*t))*w(1)*(k*w(3)+w(5))/1400000000-dep*w(4);

epsilon*w(3)-diq*w(5)-dita*w(5);

dsp*w(7)+dep*w(4)+dep*w(3)-bsp*w(6)-dpq*w(6);

dpq*w(6)-recov*w(7)-dita*w(7)+diq*w(5);

recov*w(7);

diq*w(5)+dpq*w(6);

dsp*w(7)+dep*w(4)+dep*w(3);

dita*w(5)+dita*w(7)];

[T,W]=ode45(F,[0:1:200],[S0 Sq0 E0 Eq0 I0 P0 771 34 830 1072 25]);

A7=W(:,6)';A8=W(:,2)'+W(:,4)';A9=W(:,7)';A10=W(:,9)';A11=W(:,10)';A12=W(:,8)';A13=W(:,11)';

B2=A7(1,1:1:26);B3=0.1.*A8(1,1:1:26);B4=A9(1,1:1:26);B5=A10(1,1:1:26);B6=A11(1,1:1:26);B7=A12(1,1:1:26);B8=A13(1,1:1:26);

B=[B2,B3,B4,B5,B6,B7,B8];

for o=1:182

n(o)=B(o);

end

end

save('par.mat','M')

save('qujian.mat','FangchaM')

end

%Step 2

load('Yu.mat','Yu')

for p=1:2000

PARAS=Yu(p,:);

lambda=1/14;

epsilon=1/5.2;

q=PARAS(1);

beta=PARAS(2);

c1=PARAS(3);

k=PARAS(4);

bsp=PARAS(5);

diq=PARAS(6);

dsp=PARAS(7);

dep=PARAS(8);

dpq=PARAS(9);

dita=PARAS(10);

recov=PARAS(11);

E0=PARAS(12);

I0=PARAS(13);

Eq0=PARAS(14);

S0=PARAS(15);

Sq0=PARAS(16);

c2=PARAS(17);

c3=PARAS(18);

P0=PARAS(19);

Y=[1072 1965 2684 5794 6973 9239 12167 15238 17988 19544 21558 23214 23260 24702 26359 27657 28942 23589 21675 16067 13435 10109 8969 8228 7264 6242 0.1*8420 0.1*13967 0.1*21556 0.1*30453 0.1*44132 0.1*59990 0.1*81947 0.1*102427 0.1*118478 0.1*137594 0.1*152700 0.1*171329 0.1*185555 0.1*186354 0.1*186045 0.1*189660 0.1*188183 0.1*187518 0.1*187728 0.1*185037 0.1*181386 0.1*177984 0.1*169039 0.1*158764 0.1*150539 0.1*141552 771 1208 1870 2613 4349 5739 7417 9308 11289 13748 16369 19381 22942 26302 28985 31774 33738 35982 37626 38800 52526 55748 56873 57416 57934 58016 830 1287 1975 2744 4515 5974 7711 9692 11791 14380 17205 20438 24324 28018 31161 34546 37198 40171 42638 44653 59804 63851 66492 68500 70548 72436 1072 2190 3499 7305 9382 12630 16778 21590 26609 31171 36344 41416 45387 50715 55548 59762 63678 67686 71222 74564 77371 79821 82098 84016 85579 87011 34 38 49 51 60 103 124 171 243 328 475 632 892 1153 1540 2050 2649 3281 3996 4740 5911 6723 8096 9419 10844 12552 25 41 56 80 106 132 170 213 259 304 361 425 490 563 636 722 811 908 1016 1113 1367 1380 1523 1665 1770 1868]; %

F=@(t,w)[-((1- beta)*q+ beta)*(c1+c2*exp(-c3*t))*w(1)*(k*w(3)+w(5))/1400000000-dsp*w(7)+bsp*w(6)+lambda*w(2);

(1-beta)*q*(c1+c2*exp(-c3*t))*w(1)*(k*w(3)+w(5))/1400000000-lambda*w(2);

(1-q)*beta*(c1+c2*exp(-c3*t))*w(1)*(k*w(3)+w(5))/1400000000-epsilon*w(3)-dep*w(3);

q*beta*(c1+c2*exp(-c3*t))*w(1)*(k*w(3)+w(5))/1400000000-dep*w(4);

epsilon*w(3)-diq*w(5)-dita*w(5);

dsp*w(7)+dep*w(4)+dep*w(3)-bsp*w(6)-dpq*w(6);

dpq*w(6)-recov*w(7)-dita*w(7)+diq*w(5);

recov*w(7);

diq*w(5)+dpq*w(6);

dsp*w(7)+dep*w(4)+dep*w(3);

dita*w(5)+dita*w(7)];

[T,W]=ode45(F,[0:1:200],[S0 Sq0 E0 Eq0 I0 P0 771 34 830 1072 25]);

[T2,W2]=ode45(F,[0:1:25],[S0 Sq0 E0 Eq0 I0 P0 771 34 830 1072 25]);

estp_Q=W(:,7); estp_H=W(:,9);estp_Z=W(:,11);estp_S=W(:,1);

estp_E=W(:,3); estp_Eq=W(:,4); estp_I=W(:,5); estp_P=W(:,6);

estp_R=W(:,8); estp_Y=W(:,10); estp_SqEq=W(:,2)+W(:,4);

estp_Q;estp_H;estp_Z; estp_E; estp_Eq;estp_I;estp_P;estp_R;estp_Y;estp_SqEq;

save('estp_S.mat','estp_S')

save('estp_Q.mat','estp_Q')

save('estp_H.mat','estp_H')

save('estp_Z.mat','estp_Z')

save('estp_E.mat','estp_E')

save('estp_Eq.mat','estp_Eq')

save('estp_I.mat','estp_I')

save('estp_P.mat','estp_P')

save('estp_R.mat','estp_R')

save('estp_Y.mat','estp_Y')

save('estp_SqEq.mat','estp_SqEq')

dataestp_Q(:,p)=estp_Q;dataestp_H(:,p)=estp_H;dataestp_Z(:,p)=estp_Z;dataestp_S(:,p)=estp_S;

dataestp_E(:,p)=estp_E;

dataestp_Eq(:,p)=estp_Eq;

dataestp_I(:,p)=estp_I;

dataestp_P(:,p)=estp_P;

dataestp_R(:,p)=estp_R;

dataestp_Y(:,p)=estp_Y;

dataestp_SqEq(:,p)=estp_SqEq;

dataestp_Q;dataestp_H;dataestp_Z;

dataestp_E;dataestp_Eq;dataestp_I;dataestp_P;

dataestp_R;dataestp_Y;dataestp_SqEq;

save('dataestp_S.mat','dataestp_S')

save('dataestp_Q.mat','dataestp_Q')

save('dataestp_H.mat','dataestp_H')

save('dataestp_Z.mat','dataestp_Z')

save('dataestp_E.mat','dataestp_E')

save('dataestp_Eq.mat','dataestp_Eq')

save('dataestp_I.mat','dataestp_I')

save('dataestp_P.mat','dataestp_P')

save('dataestp_R.mat','dataestp_R')

save('dataestp_Y.mat','dataestp_Y')

save('dataestp_SqEq.mat','dataestp_SqEq')

end

%Step 3 Comparison of estimated values by models (1) and (2) with the reported data

t=0:1:200;

load('par.mat','M')

load('qujian.mat','FangchaM')

load('estp_S.mat','estp_S')

load('estp_Q.mat','estp_Q')

load('estp_H.mat','estp_H')

load('estp_Z.mat','estp_Z')

load('estp_E.mat','estp_E')

load('estp_Eq.mat','estp_Eq')

load('estp_I.mat','estp_I')

load('estp_P.mat','estp_P')

load('estp_R.mat','estp_R')

load('estp_Y.mat','estp_Y')

load('estp_SqEq.mat','estp_SqEq')

load('dataestp_S.mat','dataestp_S')

load('dataestp_Q.mat','dataestp_Q')

load('dataestp_H.mat','dataestp_H')

load('dataestp_Z.mat','dataestp_Z')

load('dataestp_E.mat','dataestp_E')

load('dataestp_Eq.mat','dataestp_Eq')

load('dataestp_I.mat','dataestp_I')

load('dataestp_P.mat','dataestp_P')

load('dataestp_R.mat','dataestp_R')

load('dataestp_Y.mat','dataestp_Y')

load('dataestp_SqEq.mat','dataestp_SqEq')

S1=1.96*std(dataestp_S,0,2);

Q1=1.96*std(dataestp_Q,0,2);

H1=1.96*std(dataestp_H,0,2);

Z1=1.96*std(dataestp_Z,0,2);

E1=1.96*std(dataestp_E,0,2);

Eq1=1.96*std(dataestp_Eq,0,2);

I1=1.96*std(dataestp_I,0,2);

P1=1.96*std(dataestp_P,0,2);

R1=1.96*std(dataestp_R,0,2);

Y1=1.96*std(dataestp_Y,0,2);

SqEq1=1.96*std(dataestp_SqEq,0,2);

par1=M;par2=FangchaM;

q=par1(1);sigma_q=par2(1);

beta=par1(2);sigma_beta=par2(2);

c1=par1(3);sigma_c1=par2(3);

k=par1(4);sigma_k=par2(4);

bsp=par1(5);sigma_bsp=par2(5);

diq=par1(6);sigma_diq=par2(6);

dsp=par1(7);sigma_dsp=par2(7);

dep=par1(8);sigma_dep=par2(8);

dpq=par1(9);sigma_dpq=par2(9);

dita=par1(10);sigma_dita=par2(10);

recov=par1(11);sigma_recov=par2(11);

E0=par1(12);sigma_E0=par2(12);

I0=par1(13);sigma_I0=par2(13);

Eq0=par1(14);sigma_Eq0=par2(14);

S0=par1(15);sigma_S0=par2(15);

Sq0=par1(16);sigma_Sq0=par2(16);

c2=par1(17);sigma_c2=par2(17);

c3=par1(18);sigma_c3=par2(18);

P0=par1(19);sigma_P0=par2(19);

lambda=1/14;

epsilon=1/5.2;

c=c1+c2*exp(-c3*t);

cda=(c1+1.96*sigma_c1)+(c2+1.96*sigma_c2)*exp(-(c3-1.96*sigma_c3)*t);

cxiao=(c1-1.96*sigma_c1)+(c2-1.96*sigma_c2)*exp(-(c3+1.96*sigma_c3)*t);

s=estp_S(t+1,1)';

s1=S1(t+1,1)';

A=((1-q)*beta.*c*S0*k/(1400000000*(epsilon+dep))).^2;

B=4*(1-q)*beta.*c*S0/(1400000000*(diq+dita))*epsilon/(epsilon+dep);

R0=((1/2)*(1-q)*beta.*c*S0*k/(1400000000*(epsilon+dep))+(1/2)*sqrt(A+B)).*s./1400000000;

Ada=((1-(q-1.96*sigma_q))*(beta+1.96*sigma_beta).*cda*(S0+1.96*sigma_S0)*(k+1.96*sigma_k)/(1400000000*(epsilon+(dep-1.96*sigma_dep)))).^2;

Bda=4*(1-(q-1.96*sigma_q))*(beta+1.96*sigma_beta).*cda*(S0+1.96*sigma_S0)/(1400000000*((diq-1.96*sigma_diq)+(dita-1.96*sigma_dita)))*epsilon/(epsilon+(dep-1.96*sigma_dep));

R0da=((1/2)*(1-(q-1.96*sigma_q))*(beta+1.96*sigma_beta).*cda*(S0+1.96*sigma_S0)*(k+1.96*sigma_k)/(1400000000*(epsilon+(dep-1.96*sigma_dep)))+(1/2)*sqrt(Ada+Bda)).*(s+s1)./1400000000;

Axiao=((1-(q+1.96*sigma_q))*(beta-1.96*sigma_beta).*cxiao*(S0-1.96*sigma_S0)*(k-1.96*sigma_k)/(1400000000*(epsilon+(dep+1.96*sigma_dep)))).^2;

Bxiao=4*(1-(q+1.96*sigma_q))*(beta-1.96*sigma_beta).*cxiao*(S0-1.96*sigma_S0)/(1400000000*((diq+1.96*sigma_diq)+(dita+1.96*sigma_dita)))*epsilon/(epsilon+(dep+1.96*sigma_dep));

R0xiao=((1/2)*(1-(q+1.96*sigma_q))*(beta-1.96*sigma_beta).*cxiao*(S0-1.96*sigma_S0)*(k-1.96*sigma_k)/(1400000000*(epsilon+(dep+1.96*sigma_dep)))+(1/2)*sqrt(Axiao+Bxiao)).*(s-s1)./1400000000;

Y=[1072 1965 2684 5794 6973 9239 12167 15238 17988 19544 21558 23214 23260 24702 26359 27657 28942 23589 21675 16067 13435 10109 8969 8228 7264 6242 0.1*8420 0.1*13967 0.1*21556 0.1*30453 0.1*44132 0.1*59990 0.1*81947 0.1*102427 0.1*118478 0.1*137594 0.1*152700 0.1*171329 0.1*185555 0.1*186354 0.1*186045 0.1*189660 0.1*188183 0.1*187518 0.1*187728 0.1*185037 0.1*181386 0.1*177984 0.1*169039 0.1*158764 0.1*150539 0.1*141552 771 1208 1870 2613 4349 5739 7417 9308 11289 13748 16369 19381 22942 26302 28985 31774 33738 35982 37626 38800 52526 55748 56873 57416 57934 58016 830 1287 1975 2744 4515 5974 7711 9692 11791 14380 17205 20438 24324 28018 31161 34546 37198 40171 42638 44653 59804 63851 66492 68500 70548 72436 1072 2190 3499 7305 9382 12630 16778 21590 26609 31171 36344 41416 45387 50715 55548 59762 63678 67686 71222 74564 77371 79821 82098 84016 85579 87011 34 38 49 51 60 103 124 171 243 328 475 632 892 1153 1540 2050 2649 3281 3996 4740 5911 6723 8096 9419 10844 12552 25 41 56 80 106 132 170 213 259 304 361 425 490 563 636 722 811 908 1016 1113 1367 1380 1523 1665 1770 1868]; %

F=@(t,w)[-((1-beta)*q+ beta)*(c1+c2*exp(-c3*t))*w(1)*(k*w(3)+w(5))/1400000000-dsp*w(7)+bsp*w(6)+lambda*w(2);

(1-beta)*q*(c1+c2*exp(-c3*t))*w(1)*(k*w(3)+w(5))/1400000000-lambda*w(2);

(1-q)*beta*(c1+c2*exp(-c3*t))*w(1)*(k*w(3)+w(5))/1400000000-epsilon*w(3)-dep*w(3);

q*beta*(c1+c2*exp(-c3*t))*w(1)*(k*w(3)+w(5))/1400000000-dep*w(4);

epsilon*w(3)-diq*w(5)-dita*w(5);

dsp*w(7)+dep*w(4)+dep*w(3)-bsp*w(6)-dpq*w(6);

dpq*w(6)-recov*w(7)-dita*w(7)+diq*w(5);

recov*w(7);

diq*w(5)+dpq*w(6);

dsp*w(7)+dep*w(4)+dep*w(3);

dita*w(5)+dita*w(7)];

[T,W]=ode45(F,[0:1:200],[S0 Sq0 E0 Eq0 I0 P0 771 34 830 1072 25]);

[T2,W2]=ode45(F,[0:1:25],[S0 Sq0 E0 Eq0 I0 P0 771 34 830 1072 25]);

tmax=200;

figure(1) % S(t)

plot(T,W(:,1),'k-','LineWidth',4);

xlabel('Days from January 23, 2020');

ylabel('Number of free susceptible people');

figure(2) % Sq(t)

plot(T,W(:,2),'k-','LineWidth',4);

xlabel('Days from January 23, 2020');

ylabel('Number of traced susceptible people');

figure(3) % E(t)

plot([0:1:tmax],estp_E,'k-',[0:1:tmax],estp_E+E1,'r-',[0:1:tmax],estp_E-E1,'g-','LineWidth',3)

xlabel('Days from January 23, 2020');

ylabel('Number of free latent people');

figure(4) % Eq(t)

plot([0:1:tmax],estp_Eq,'k-',[0:1:tmax],estp_Eq+Eq1,'r-',[0:1:tmax],estp_Eq-Eq1,'g-','LineWidth',3)

xlabel('Days from January 23, 2020');

ylabel('Number of traced latent people');

figure(5) % I(t)

plot([0:1:tmax],estp_I,'k-',[0:1:tmax],estp_I+I1,'r-',[0:1:tmax],estp_I-I1,'g-','LineWidth',3)

xlabel('Days from January 23, 2020');

ylabel('Number of free infectious people');

figure(6) % R(t)

plot([0:1:tmax],estp_R,'k-',[0:1:tmax],estp_R+R1,'r-',[0:1:tmax],estp_R-R1,'g-','LineWidth',3)

hold on

plot([0 1 2 3 4 5 6 7 8 9 10 11 12 13 14 15 16 17 18 19 20 21 22 23 24 25],[34 38 49 51 60 103 124 171 243 328 475 632 892 1153 1540 2050 2649 3281 3996 4740 5911 6723 8096 9419 10844 12552],'bo','LineWidth',2,'MarkerSize',9);

xlabel('Days from January 23, 2020');

ylabel('Number of recovered cases');

figure(7) % H(t)

plot([0:1:tmax],estp_H,'k-',[0:1:tmax],estp_H+H1,'r-',[0:1:tmax],estp_H-H1,'g-','LineWidth',3)

hold on

plot([0 1 2 3 4 5 6 7 8 9 10 11 12 13 14 15 16 17 18 19 20 21 22 23 24 25],[830 1287 1975 2744 4515 5974 7711 9692 11791 14380 17205 20438 24324 28018 31161 34546 37198 40171 42638 44653 59804 63851 66492 68500 70548 72436],'bo','LineWidth',2,'MarkerSize',9);

xlabel('Days from January 23, 2020');

ylabel('Cumulative number of confirmed cases');

figure(8) % Q(t)

plot([0:1:tmax],estp_Q,'k-',[0:1:tmax],estp_Q+Q1,'r-',[0:1:tmax],estp_Q-Q1,'g-','LineWidth',3)

hold on

plot([0 1 2 3 4 5 6 7 8 9 10 11 12 13 14 15 16 17 18 19 20 21 22 23 24 25],[771 1208 1870 2613 4349 5739 7417 9308 11289 13748 16369 19381 22942 26302 28985 31774 33738 35982 37626 38800 52526 55748 56873 57416 57934 58016],'bo','LineWidth',2,'MarkerSize',9);

xlabel('Days from January 23, 2020');

ylabel('Number of existing confirmed cases');

figure(9) % P(t)

plot([0:1:tmax],estp_P,'k-',[0:1:tmax],estp_P+P1,'r-',[0:1:tmax],estp_P-P1,'g-','LineWidth',3)

hold on

plot([0 1 2 3 4 5 6 7 8 9 10 11 12 13 14 15 16 17 18 19 20 21 22 23 24 25],[1072 1965 2684 5794 6973 9239 12167 15238 17988 19544 21558 23214 23260 24702 26359 27657 28942 23589 21675 16067 13435 10109 8969 8228 7264 6242],'bo','LineWidth',2,'MarkerSize',9);

xlabel('Days from January 23, 2020');

ylabel('Number of existing suspected cases');

figure(10) % Sq(t)+Eq(t)

plot([0:1:tmax],estp_SqEq,'k-',[0:1:tmax],estp_SqEq+SqEq1,'r-',[0:1:tmax],estp_SqEq-SqEq1,'g-','LineWidth',3);

hold on

plot([0 1 2 3 4 5 6 7 8 9 10 11 12 13 14 15 16 17 18 19 20 21 22 23 24 25],[8420 13967 21556 30453 44132 59990 81947 102427 118478 137594 152700 171329 185555 186354 186045 189660 188183 187518 187728 185037 181386 177984 169039 158764 150539 141552],'bo','LineWidth',2,'MarkerSize',9);

xlabel('Days from January 23, 2020');

ylabel('Close contacts under medical observation');

figure(11) %Z(t)

plot([0:1:tmax],estp_Z,'k-',[0:1:tmax],estp_Z+Z1,'r-',[0:1:tmax],estp_Z-Z1,'g-','LineWidth',3);

hold on

plot([0 1 2 3 4 5 6 7 8 9 10 11 12 13 14 15 16 17 18 19 20 21 22 23 24 25],[25 41 56 80 106 132 170 213 259 304 361 425 490 563 636 722 811 908 1016 1113 1367 1380 1523 1665 1770 1868],'bo','LineWidth',2,'MarkerSize',9);

xlabel('Days from January 23, 2020');

ylabel('Cumulative number of deaths');

figure(12) % Y(t)

plot([0:1:tmax],estp_Y,'k-',[0:1:tmax],estp_Y+Y1,'r-',[0:1:tmax],estp_Y-Y1,'g-','LineWidth',3);

hold on

plot([0 1 2 3 4 5 6 7 8 9 10 11 12 13 14 15 16 17 18 19 20 21 22 23 24 25],[1072 2190 3499 7305 9382 12630 16778 21590 26609 31171 36344 41416 45387 50715 55548 59762 63678 67686 71222 74564 77371 79821 82098 84016 85579 87011],'bo','LineWidth',2,'MarkerSize',9);

xlabel('Days from January 23, 2020');

ylabel('Cumulative number of suspected cases');

figure(13) % R0(t)

plot([0:1:200],R0,'k-',[0:1:200],R0da,'r--',[0:1:200],R0xiao,'g--')

xlabel('Days from January 23, 2020');

ylabel('Effective reproduction number');

legend('Estimated values by model','95% CI','95% CI');

set(gca,'linewidth',2)

**References**

1. National Health Commission of the People’s Republic of China. Daily report about SARS-CoV-2. <http://www.nhc.gov.cn/xcs/yqtb/list_gzbd.shtml> [Accessed 17 Feb 2020]
2. Health Commission of Hubei Province. Daily report about SARS-CoV-2. <http://wjw.hubei.gov.cn/fbjd/dtyw/index.shtml> [Accessed 17 Feb 2020]
3. Wuhan Municipal Health Commission. Daily report about SARS-CoV-2. <http://wjw.wuhan.gov.cn/front/web/list2nd/no/710> [Accessed 17 Feb 2020]
